# From Atomic‐Scale Engineering to Industry‐Ready Systems: Prospects for Seawater Electrolysis Toward Hydrogen

**DOI:** 10.1002/smll.75190

**Published:** 2026-08-14

**Authors:** Jiaxi Ding, Yutong Wu, Yanzhe Li, Jodie A Yuwono, Joshua Zheyan Soo, Shenlong Zhao, Chuan Zhao, Siva Karuturi, Doudou Zhang

**Affiliations:** ^1^ School of Engineering College of Systems and Society The Australian National University Canberra Australia; ^2^ School of Chemistry The University of New South Wales Sydney Australia; ^3^ CAS Key Laboratory of Nanosystem and Hierarchical Fabrication CAS Center for Excellence in Nanoscience National Center for Nanoscience and Technology Beijing China; ^4^ Faculty of Sciences Engineering and Technology School of Chemical Engineering University of Adelaide Adelaide Australia; ^5^ Department of Chemical Engineering School of Engineering Monash University Malaysia Jalan Lagoon Selatan Bandar Sunway Malaysia; ^6^ Center for Net‐Zero Technology Monash University Malaysia Jalan Lagoon Selatan Bandar Sunway Malaysia; ^7^ School of Engineering Macquarie University Sydney Australia

**Keywords:** DFT, direct seawater electrolysis, electrocatalysts, electrolyzers

## Abstract

To accelerate the industrial application of hydrogen (H_2_) and support the global energy transition, seawater electrolysis emerges as a promising strategy to address energy demands and freshwater scarcity. Owing to its natural abundance and high ionic conductivity, seawater represents an attractive electrolyte resource. However, its complex chemical composition introduces significant challenges, including sluggish kinetics, catalyst poisoning, chlorine evolution, membrane degradation, and long‐term corrosion. Recent years have witnessed significant progress in electrocatalyst development, electrolyzer design, and mechanistic understanding aimed at overcoming these seawater‐specific constraints. In this review, we provide a comprehensive and critical overview of advances in seawater electrolysis, covering electrocatalyst design strategies for the hydrogen evolution reaction, oxygen evolution reaction, and bifunctional reactions, as well as developments in device architectures and operations. Emphasis is placed on scalable synthesis, stability under real seawater conditions, and theoretical insights from density functional theory calculations into reaction pathways and degradation mechanisms. In addition, key challenges related to standardized testing protocols, system integration with renewable energy sources, and techno‐economic viability are discussed. Finally, future perspectives are outlined to guide the design of durable, scalable, and economically feasible seawater electrolysis systems, highlighting critical research directions to accelerate the transition toward large‐scale green H_2_ production.

## Graphical abstract

Seawater electrolysis is emerging as a viable route for large‐scale green hydrogen production, yet faces challenges from complex chemistries and harsh operating conditions. This review critically examines advances in electrocatalysts, device structures, and mechanistic understanding, highlighting scalable design strategies, durability in real seawater, and theoretical insights, while outlining key challenges and future directions toward industrial deployment.

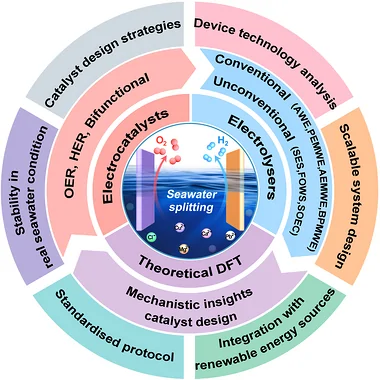

## 1 Introduction

The ongoing global transition from carbon‐intensive fossil fuels to renewable energy sources highlights the need for hydrogen (H_2_), as it is considered the most promising alternative. The high energy density of H_2_ makes it suitable for a wide range of applications, and its zero‐emission usage aligns with the current context of carbon neutrality [1, 2, 3]. Among the different ways of H_2_ generation, electrochemical water electrolysis has great potential because it produces H_2_ of exceptional purity and the process doesn't involve carbon emissions, unlike the traditional way via methane reforming [4]. However, the increasingly scarce freshwater resources may pose a hindrance to the large‐scale industrial application of water electrolysis. Many coastal locations that have abundant renewable energy sources like solar or wind to generate electricity may not be suitable for water electrolysis plants due to the lack of freshwater. Meanwhile, seawater makes up more than 96% of the global water resources and is considered the most abundant aqueous electrolyte reserve [5, 6]. Under this circumstance, seawater electrolysis is an excellent alternative to pure water splitting technologies [5, 7].

Compared with conventional freshwater electrolysis, seawater electrolysis presents a uniquely advantageous yet technically demanding pathway. While freshwater electrolytes exhibit low intrinsic conductivity and require additives of alkaline or acid, seawater naturally provides a high conductivity of 33.9 mS/cm at neutral pH, reducing the need for electrolyte modification and lowering the additional corrosion burden associated with added acids or alkaline [8, 9]. However, despite these advantages, practical seawater splitting still faces substantial obstacles. The complex composition of seawater introduces challenges not encountered in freshwater systems. Dissolved ions and impurities can introduce sluggish reaction kinetics, catalyst poisoning, and membrane contamination [10, 11], while the high chloride ion (Cl^−^) concentration promotes chloride oxidation reaction (ClOR), competing with oxygen evolution reaction (OER) and compromising Faradaic efficiency (FE) and catalyst stability [12, 13, 14, 15]. In addition, alkaline earth metal ions may precipitate with OH^−^ near the cathode, hindering hydrogen evolution reaction (HER) activity by blocking active sites [16, 17, 18]. These seawater‐specific issues highlight why system design for seawater electrolysis requires fundamentally different considerations than those applied to freshwater systems. Overcoming these barriers is essential for developing practical seawater electrolyzers capable of supporting large‐scale H_2_ production for sustainable energy technologies.

Seawater electrolysis, compared with coupling seawater reverse osmosis (RO) and freshwater electrolysis, offers advantages in terms of simplicity. Although some techno‐economic analyses have concluded that seawater electrolysis is currently less economically viable than the alternative [19], it is important to note that the direct integration of seawater RO with conventional electrolysis is not yet technologically feasible. This limitation arises from the residual impurities in RO‐treated water and the insufficient capacity of seawater RO to meet the projected rapidly growing H_2_ production demands of large‐scale electrolysis systems [20, 21]. Moreover, recent advances in catalysts and cell design have enabled seawater electrolysis to operate without the need for desalination, alkalization, or acidification steps [22, 23]. The simplification in engineering that eliminates the cost of seawater pre‐treatment, cost of additives, and other maintenance costs could significantly improve the economic feasibility of seawater splitting, reducing major costs predominantly to system installation [24, 25].

The rapid growth of seawater electrolysis has been accompanied by the increasing number of reviews. After examining the published literature on seawater splitting, we found that it is very necessary to have a comprehensive overview of seawater electrolysis that integrates electrocatalyst materials and design strategies, device and technology advances, and theoretical insights into reaction mechanisms to help researchers interested in this field. The reviews recently published have provided valuable accounts of catalyst performance, competing chlorine chemistry, and emerging cell designs [26, 27, 28]. Unlike previous reviews that primarily summarize catalyst compositions [27, 29, 30], this review establishes a comprehensive framework that connects atomic‐scale catalyst engineering with industrial seawater electrolysis and, most importantly, incorporates mechanism insights from computational methods. Section 2 first defines the critical performance metrics and identifies the fundamental bottlenecks limiting industrial implementation, providing a clear benchmark for catalyst and system development. Section 3 systematically compares alkaline, neutral, and acidic seawater electrolysis while distinguishing the unique scientific and engineering challenges arising from complex seawater chemistry compared with freshwater electrolysis. Building on these challenges, Section 4 elucidates the atomic origins of catalyst performance by correlating active‐site composition, electronic structure, and interfacial reaction microenvironments with activity, selectivity, and corrosion resistance. Section 5 extends this understanding beyond catalyst design to practical device integration, evaluating how atomically engineered catalysts perform in different electrolyzer architectures, membrane configurations, and emerging pilot‐scale demonstrations. Importantly, Section 6 introduces theoretical and computational perspectives, integrating density functional theory, machine learning, and multiscale modelling to bridge fundamental understanding with rational catalyst discovery and device optimization. By linking industrial requirements, atomic‐level design principles, practical electrolyzer engineering, and predictive computational approaches within a single framework, this review moves beyond a conventional literature survey and provides a roadmap for translating fundamental catalyst innovations into commercially viable seawater electrolysis technologies.

## 2 Principles of Seawater Splitting

### 2.1 Metrics

Seawater electrolysis is evaluated using a range of metrics that reflect its efficiency, operational performance, and environmental impact. These metrics provide important benchmarks for assessing the feasibility and practical potential of this technology for H_2_ production.

The OER is a complex four‐electron process that involves the adsorption and dissociation of OH^−^, formation of O–O intermediates, and eventual release of O_2_. Due to the much higher OH^−^ concentration in alkaline media compared with neutral seawater, the reaction proceeds significantly more slowly under neutral conditions [31]. Moreover, in DSE, the presence of Cl^−^ leads to harmful side reactions at the anode. ClOR corrodes the OER catalyst and reduces the electrolyzers’ lifespan [5, 9]. Consequently, effective OER catalysts must combine low overpotential, high selectivity, and strong resistance to corrosion to ensure stable performance over extended operation in the seawater environment.

The HER occurs at the cathode. Although it is thermodynamically more favorable and mechanistically simpler than OER, comparatively less attention has been devoted to HER electrode development. This is partially because OER competes with ClOR, whose thermodynamic potentials are very close, making anode selectivity a major research focus. Nevertheless, HER in DSE faces significant challenges, including Mg^2+^ and Ca^2+^, small particles, and microorganisms, all of which can accumulate on catalyst surfaces, block active sites, reduce catalytic activity, and limit durability [20, 32]. Particularly, in neutral electrolytes, local pH at the cathode can increase during operation, promoting the precipitation of Mg(OH)_2_ and Ca(OH)_2_. Under neutral seawater conditions, such precipitation has been reported to reduce the HER current by half [33]. The evaluation metrics for HER in DSE are generally similar to those for OER, including catalytic activity, long‐term stability, selectivity, and resistance to corrosion. However, particular emphasis must be placed on resistance to contamination and precipitation.

Despite the costless and infinite seawater resources, low energy efficiency is one of the major factors hindering the large‐scale DSE deployment. The energy efficiency is inherently limited by the large thermodynamic potential required by the anodic OER and the sluggish kinetics associated with its multi‐step proton‐coupled electron transfer process. In addition, the competing ClOR at the anode can significantly influence the energy efficiency of DSE [34]. Improving energy efficiency from a kinetic perspective requires strategies that reduce the operating voltage while simultaneously enhancing FE [35]. The purity of the produced H_2_ is an important factor influencing the overall energy efficiency [36].

Another important metric for evaluating DSE is its economic viability. In techno‐economic analysis (TEA) of DSE, the levelized cost of Hydrogen (LCOH) is one of the most widely used indicators and serves as a benchmark for assessing the cost‐effectiveness of different H_2_ production pathways [37]. Although TEA is not the primary focus of this review, reducing LCOH remains the implicit objective driving the development of catalysts and electrolyzers. During the calculation of LCOH, it is obvious that DSE faces challenges in both capital and operational expenditure. The initial cost of DSE can exceed twice that of conventional water electrolysis systems. In addition, the complex composition of seawater and the corrosive operating environment significantly increase operational costs, as membranes, catalysts, and other system components may require frequent replacement [37].

### 2.2 Challenges of DSE

The composition of seawater is very complicated. The major dissolved ions are Na^+^ and Cl^−^ of concentration close to 0.6 M. Other salts and elements also exist in lower concentration, such as Mg^2+^, K^+^, CO_3_2^−^, SO_4_^2−^, and S [21]. The complexity of composition has both advantages and disadvantages. The benefit is the increased salinity and the resultant higher conductivity. Zheng et al. [38] collected seawater from different depths of the mid‐low latitudes of the Pacific and Indian oceans for six consecutive years and concluded that the seawater conductivity falls within the range of 3–6 S/m at every location. It is noteworthy that salinity is not the only factor of conductivity; the synergistic effect of salinity with temperature and pressure determines the conductivity of seawater.

**FIGURE 1:**
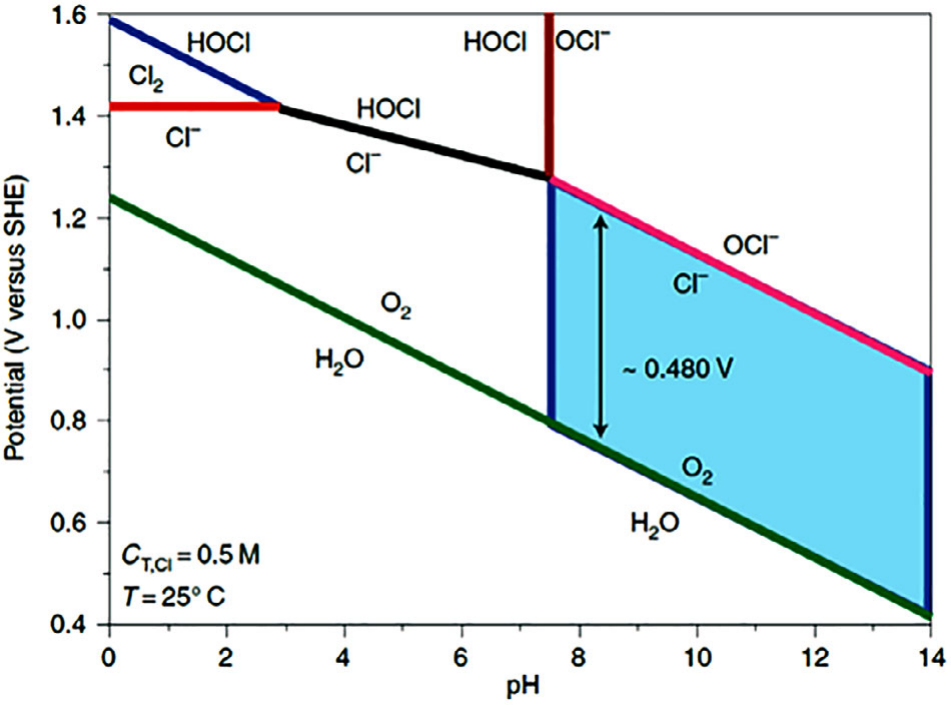
Pourbaix diagram of competing OER and chloride chemistry model in simulated seawater with 0.5 m NaCl. Reproduced with permission [11]. Copyright 2016, Wiley‐VCH.

The downside of the composition is the reactions of these impurities that can happen at the electrodes. One of the biggest problems is the aforementioned ClOR that competes with OER at the anode. The large amounts of Cl^−^ in the seawater are oxidized and generate Cl_2_ [5, 12, 39]. To assess the extent of ClOR, a Pourbaix diagram can be applied, as shown in Figure 1. While the theoretical corrosion requirement for seawater electrolysis is low, practical applications demand catalysts that can endure the highly corrosive marine environment. A key challenge is the oxidative attack from Cl^−^, particularly at the anode. Therefore, catalysts must exhibit strong resistance to Cl_2_ and Cl^−^ poisoning [17, 40, 41]. These concerns are especially pronounced under alkaline conditions, which are often preferred to suppress ClOR but impose more severe anti‐corrosion requirements on catalyst surfaces [21, 42, 43, 44].

Beyond corrosion concerns, neutral seawater conditions introduce additional electrochemical challenges. The local pH increases near the cathode during HER can still induce corrosion, while the lack of reactant ions H^+^ and OH^−^ raises the energy barrier for both HER and OER [15, 31, 45]. To address this, many studies alkalize the seawater. For example, the bifunctional CoSe_2_ nanocrystals embedded into a carbon framework required 134 and 235 mV for HER and OER at 10 mA/cm^2^ in alkalized natural seawater (with 1 m KOH). The voltage was 1.842 V at 500 mA/cm^2^ for two‐electrode seawater splitting [46]. There are also cases where researchers use highly concentrated alkaline solutions for more complete suppression of ClOR. Wang et al. treated seawater with KOH of 9.5 m. Another advantage of the treatment is that the Mg^2+^ and Ca^2+^ were precipitated, ensuring that they didn't affect the stability of the electrolysis process. Their OER catalyst NiCoFe‐B_i_ required only 267 mV at 500 mA/cm^2^ and remained stable for 1000 h [47]. Another notable approach involves designing catalysts with intrinsic selectivity against ClOR. Silviya's team ran a series of tests and computational calculations to determine the selective characteristics of the Co‐B catalysts for OER over ClOR. Their Co‐B catalyst exhibited 182 and 305 mV for HER and OER at 10 mA/cm^2^. The selectivity was explored by measuring the FE, which was 99.5% for HER and initially 96% for OER. The o‐tolidine test also confirmed that no chloride product was detected [48].

The by‐products caused by ClOR include not only Cl_2_, but also highly active hypochlorite (OCl^−^) ions. When pH < 3, Equation (1) represents the chlorine evolution reaction (ClER) that predominates over Equation (2). Hypochlorite formation takes place at a pH of 3–7.5 [49]. Even though there have been multiple ways to prevent the occurrence of ClER, the complex chemical environment of seawater makes complete avoidance difficult [12].

Although the OER is thermodynamically more favorable than the ClOR, the ClOR proceeds through a two‐electron pathway and therefore exhibits faster kinetics than the four‐electron OER process, as shown by Equations (1) and (2). In alkaline media, a stable potential difference of approximately 480 mV exists between the OER and ClOR, which enables selective seawater electrolysis. Consequently, to suppress ClOR, the overpotential of OER should be kept below 480 mV even at high current densities [50].

(1) $$2Cl^{-}\to Cl_{2}+2e^{-}\left(E^{\theta }=1.36V,acidicconditions\right)$$

Hypochlorite formation:

$$Cl^{-}+2OH^{-}\to ClO^{-}+H_{2}O+2e^{-}\;\left(\;E^{\theta }=0.89V,alkalineconditions\right)$$

Or

(2) $$Cl^{-}+H_{2}O\to HClO+H^{+}+2e^{-}$$

In alkaline electrolytes, Cl^−^‐induced oxidation of metal cations leads to the formation of metal chloride–hydroxide species, causing corrosion of OER catalysts (Equations ((2), (3), (4))). In seawater, this pathway significantly accelerates degradation of active sites and current collectors. This corrosion process follows a three‐step mechanism: polarization, dissolution, and hydroxide formation. Under high anodic potential, the highest occupied molecular orbital–lowest unoccupied molecular orbital energy levels of the anode are lowered, promoting the adsorption of Cl^−^ ions onto the positively charged active surface. In the absence of a protective passive layer, these aggressive anions coordinate with the surface‐adsorbed ions, leading to the dissolution of soluble metal chlorides. Subsequently, in an alkaline environment, hydroxide ions (OH^−^) facilitate the conversion of metal chloride species into their respective hydroxides. This process gradually depletes OH^−^, lowering the local pH and further accelerating corrosion. The sustained deterioration of transition metal‐based catalysts due to Cl^−^‐induced corrosion ultimately leads to catalyst poisoning and diminished catalytic stability [51].

Adsorption

(3) $$Cl^{-}+M\to MCl_{ads}+e^{-}$$

Dissolution

(4) $$MCl_{ads}+Cl^{-}\to MCl_{x}^{-}$$

Hydroxide formation

(5) $$MCl_{x}^{-}+OH^{-}\to M{\left(OH\right)}_{x}+Cl^{-}$$

Hence, enhancing the corrosion resistance of electrodes is essential to prevent the formation of corrosive and toxic Cl_2_ during the electrolysis of seawater. This can be addressed through strategies such as developing selective OER catalysts to suppress undesired side reactions [25, 52], or incorporating a layer, such as a transition metal sulfide layer, that effectively suppresses the Cl^−^ adsorption utilizing electrostatic repulsion [53].

Beyond chloride chemistry, the cathode faces a distinct fouling problem. During HER, water reduction consumes protons and raises the local pH at the cathode surface, driving the precipitation of insoluble Mg(OH)_2_ and Ca(OH)_2_ from Mg^2+^ and Ca^2+^, hindering the stable performance of HER significantly [16]. The HER can increase the pH of the environment of the cathode abruptly and result in the formation of precipitation [54]. The precipitation generated on the surface of the cathodic electrode can decrease the current density by 50% [55]. The precipitation is a progressive and cumulative process. Under continuous operation, the deposits build up on the electrode and within the cell, ultimately requiring the system to be shut down for removal of fragments or replacement of degraded cell components, which directly undermines the uninterrupted operation required at industrial scale [56].

Electrode degradation in DSE extends well beyond the anodic chlorine chemistry and the cathodic precipitation buildup. Despite its far lower concentration, Br^−^ can drive broad‐area corrosion, result in pitting, delaminate the catalyst layer from Ni‐based substrates, and cause a rapid loss of OER performance [57]. Sulfate ions (SO_4_^2^^−^) have also been shown to accelerate the corrosion of metallic catalyst substrates by destabilizing the surface, promoting the formation of metal hydroxides or chlorides rather than maintaining a stable surface chemistry. The SO_4_^2^^−^ ions go through the cyclic release/restart process and result in electrode degradation [40].

Except for electrodes, other electrolyzer components present challenges of their own, most notably the membrane. Membrane degradation arises through two distinct pathways, chemical and physical. Chemical degradation involves nucleophilic substitution, elimination, phenyl oxidation, and methyl and/or proton rearrangement reactions of the ionomer. Physical degradation is attributed to precipitation or blockage [26].

Among the obstacles to industrializing seawater splitting, durability rather than activity is a more challenging constraint. The complex components of seawater deactivate electrocatalysts and drive severe corrosion, so although DSE is in principle scalable and economically attractive where freshwater is costly or impractical to supply, its translation to industrial‐scale hydrogen generation has not yet been realized [58]. The durability issue is most serious at the system level, especially since membrane electrolyzers have significantly inferior durability in DSE, compared to freshwater electrolyzers. Realizing continuous, industrial‐scale operation hinges on extending the operational lifetime of catalysts, interfaces, and membranes under realistic seawater conditions [58].

Collectively, the challenges outlined above pose significant barriers to the practical implementation of DSE, including catalyst corrosion, low energy efficiencies and FE, competing side reactions, and high operational costs. Addressing these obstacles requires approaches from different aspects, such as developing robust and selective electrocatalysts, engineering corrosion‐resistant materials, and electrolyte modification. A range of engineering strategies has emerged in response. At the catalyst and electrode level, these include electronic‐structure modulation, corrosion‐resistant passivation layers, and anion‐selective or Lewis‐acid layers that repel Cl^−^ and sustain a locally alkaline microenvironment [59, 60]. At the device and system level, these include pH‐asymmetric electrolyzers with cation‐selective membranes [61], membrane‐based in situ desalination, and desalination‐integrated configurations that intercept Cl^−^, Ca^2+^, and Mg^2+^ before they reach the active sites [60]. The catalyst‐level strategies are discussed in Section 4, and the device‐level strategies in Section 5.

## 3 Current State of Seawater Electrolysis

### 3.1 Technological Maturity and Practical Implementations

**FIGURE 2:**
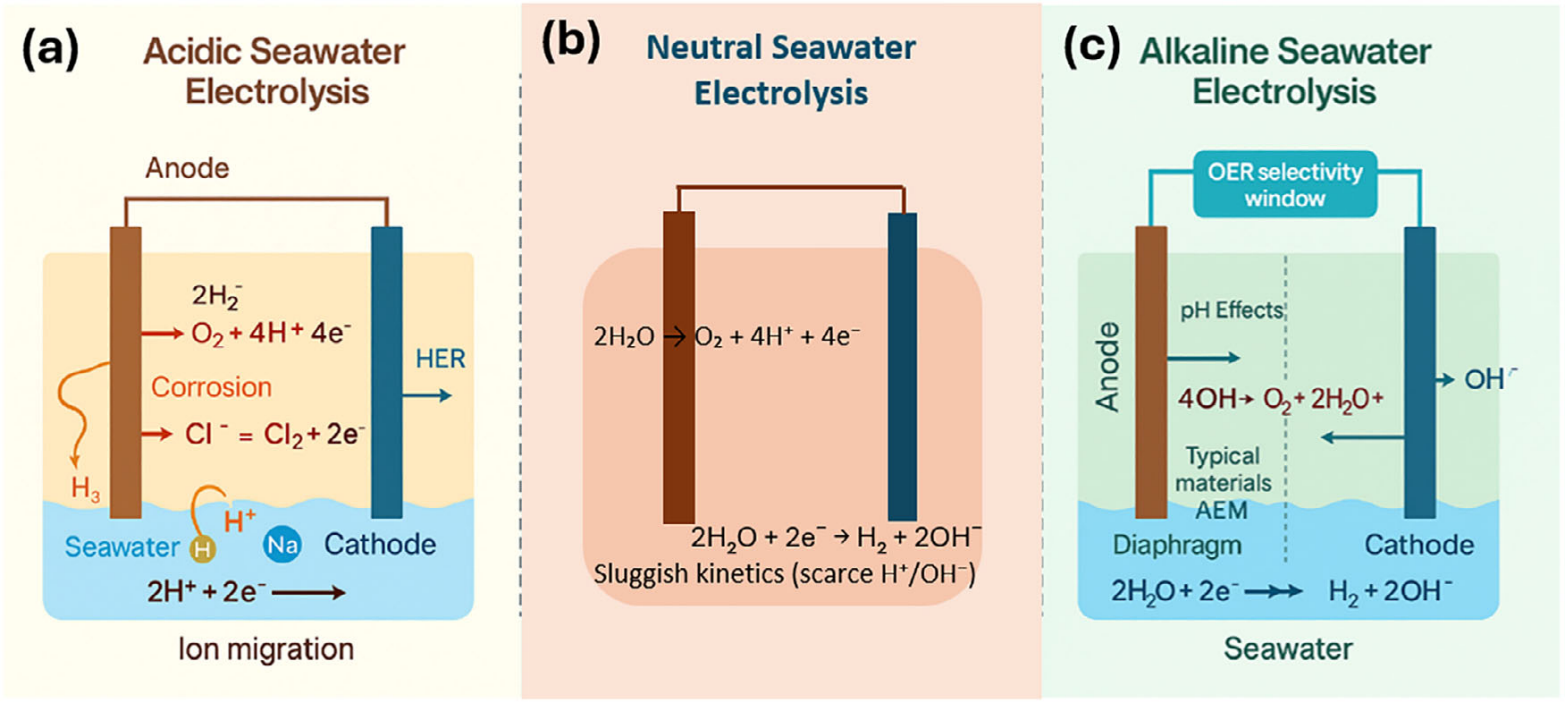
(a) Acidic [64], (b) pH‐neutral, and (c) alkaline [64] seawater electrolysis mechanisms. (a,c) Reproduced under terms of the CC‐BY license [64]. Copyright 2025, Elsevier.

Seawater electrolysis is gaining momentum as a sustainable H_2_ production pathway, but its practical implementation remains limited by challenges such as chloride‐induced corrosion, salt precipitation, and the need for long‐term system stability. To better evaluate the maturity of DSE technologies, a common evaluation framework has been established to benchmark technological progress. For commercial viability, DSEs are generally expected to sustain industrial current density over 1 A/cm^2^, cell voltages below ∼2 V, with stack lifetimes approaching 60 000 h [19, 62]. Relative to freshwater operation, seawater typically incurs a higher cell voltage at a given current density owing to greater ionic resistance, competing chloride chemistry, and additional overpotentials, which together increase the energy consumption to meet the industrial targets [63]. This section reviews recent pilot‐scale demonstrations and technological developments under acidic, neutral, and alkaline conditions, as shown in Figure 2, the mechanisms of DSEs under conditions of these three conditions are varied.

#### 3.1.1 Acidic Seawater Electrolysis Systems

In acidic systems, proton exchange membrane (PEM) electrolyzers have demonstrated high efficiency but require purified water to avoid chloride corrosion. The Sealhyf project presented a notable offshore pilot‐scale application, deploying a 1 MW PEM electrolyzer that produced up to 400 kg of H_2_ per day using seawater as the source. However, the system included a desalination unit upstream, indicating that direct seawater electrolysis remained unfeasible for conventional PEM technology due to its vulnerability to Cl^−^ and Cl_2_ [65]. A more promising approach was demonstrated by Marin et al., who developed a bipolar membrane electrolyzer (BPMWE) capable of operating on seawater. The BPM created asymmetric pH conditions, suppressing ClOR at the anode and controlling chloride FE as low as 0.005%, compared to 10% in standard PEM cells. This enabled about 140 times longer operation without significant degradation, highlighting the potential of BPM configurations to improve durability in acidic seawater electrolysis [66]. Li et al. reported a pretreatment‐free BPM system in which an ultrathin cation‐exchange layer and a catalytic MXene@FeOOH interlayer cooperatively block chloride crossover, suppressing the ClER, while protons generated at the BPM junction maintain a mildly acidic catholyte that prevents Mg(OH)_2_ and Ca(OH)_2_ scaling. The hydrogen FE of this device remained at ∼100% for 80 h at 100 mA/cm^2^ in real seawater configuration [67].

#### 3.1.2 Neutral pH Direct Seawater Electrolysis Systems

Neutral seawater electrolysis poses significant challenges due to low concentrations of reactive ions and increased susceptibility to corrosion and scaling. At the near‐neutral pH (∼8) of natural seawater, the low concentrations of free H^+^ and OH^−^ result in sluggish kinetics at both electrodes [68]. Nevertheless, several successful demonstrations have emerged. Liu's team reported the first offshore floating electrolyzer powered by wind and directly fed with untreated seawater. Operated in Xinghua Bay, the system maintained stable performance for 240 h, producing high‐purity H_2_ at 250 mA/cm^2^ with cell voltages around 2.14 V, comparable to onshore systems [69]. Shi's group developed a sodium‐ion‐conducted asymmetric electrolyzer that can prevent both corrosion and precipitation using natural seawater as electrolyte. The system exhibited exceptional performance with voltages as low as 1.66 V to reach 400 mA/cm^2^ at 80°C. It also reached an estimated cost of $1.36/kg H_2_, meeting the US DOE's 2025 target [3]. Dresp et al. reported an asymmetric cell configuration that circulates neutral seawater at the cathode, which enhances the DSE performance and avoids seawater alkalinization. Operating at 200 mA/cm^2^ from an initial 1.7 V, the cell drifted only ∼8–10 mV per 12 h, accumulating roughly 100 mV over 100 h. Despite the slight increase of voltage, the oxygen FE remained identical throughout the measurement [70]. These examples show that neutral seawater electrolysis is technically viable, but current systems remain limited to small‐scale and short‐term operation.

#### 3.1.3 Alkaline Seawater Electrolysis Systems

Alkaline seawater electrolysis has attracted considerable attention due to its compatibility with robust, non‐precious metals‐based catalysts and mature cell configurations. Xie et al. developed a laser‐induced high‐entropy alloy (HEA) bifunctional catalyst capable of sustaining 250 mA/cm^2^ for over 3000 h with a negligible voltage increase in the alkaline seawater electrolyzer [71]. Sun's group demonstrated a NiFe anode that enabled an alkaline seawater electrolyzer to operate stably at 1000 mA/cm^2^ for over 40 days [72]. Another strategy reported by Cheng et al. modifies the electrolyte by adding F^−^ additive. The approach was shown to substantially improve NiFe‐layered double hydroxide (LDH) anode performance in alkaline seawater. The system with optimized F^−^ concentration has demonstrated stable operation at 2 A/cm^2^ for 1000 h in alkaline seawater electrolyte [73]. The anodic activity was enhanced significantly because of F^−^ adjusted the electronic environment of Ni and Fe due to its high electronegativity and charge density, which resulted in improved OH^−^ adsorption. These studies suggest that alkaline systems are nearing pilot‐readiness, especially when enhanced with chloride‐tolerant bifunctional materials.

### 3.2 Performance Comparison: Freshwater Versus Seawater Electrolysis

To assess the current adaptability of electrolyzers for practical seawater splitting, many studies evaluate systems using the same catalyst and device while varying only the electrolyte between freshwater and seawater. A summary of these studies is presented in the following comparison table, highlighting the performance differences observed under the two operating conditions. Table 1 summarizes key metrics, including catalytic activity, stability, and specific strategies employed to enhance corrosion resistance. These examples highlight how structural engineering, surface modification, and phase control contribute to maintaining efficiency and durability under harsh seawater conditions.

**TABLE 1:** Comparison of Electrolysis Performance in fresh water and seawater electrolysis.

Catalysts HER//OER	Activity		Stability		Strategy to enhance corrosion resistance	Refs.
	Freshwater	Seawater	freshwater	Seawater		
Ni_2_P‐Fe_2_P // Ni_2_P‐Fe_2_P	(1 m KOH) 1.682, 1.865 V at 100, 500 mA/cm^2^	(1 m KOH seawater) 1.811 and 2.004 V at 100 and 500 mA/cm^2^.	3000 CV cycles and remained stable for 24 h sequentially at 100 and 500 mA/cm^2^	could work continuously for 36 h at 100 mA/cm^2^ and for more than 23 h at 500 mA/cm^2^	P alloying reduces the thermodynamic tendency of metal dissolution, thereby improving corrosion resistance and chemical stability	[74]
Ru‐MOF Ni*_x_* Fe_1–_ *_x_* Se_2_/NF // Ru‐MOF Ni*_x_* Fe_1–_ *_x_* Se_2_/NF	(1 m KOH) OER: 250 and 290 mV at 100 and 500 mA/cm^2^ HER: 130 and 199 V at 100 and 500 mA/cm^2^ 1.61 V at 100 mA/cm^2^	(1 m KOH + natural seawater) OER: 310, 390 mV at 100 500 mA/cm^2^, HER: 189 315 mV at 100 500 mA/cm^2^. 1.71 V at 100 mA/cm^2^	(1 m KOH) 100 h at 100 and 500 mA/cm^2^	(1 m KOH + natural seawater) 100 h at 100 and 500 mA/cm^2^	During HER, partial conversion of metal selenides into in situ formed NiOOH and FeOOH creates a protective oxyhydroxide shell, which serves as active catalytic sites and enhances corrosion resistance. Additionally, the absence of OCl^−^ formation during OER indicates high selectivity and resistance to chloride‐induced degradation.	[75]
FeCo_2_O_4_‐FeCo_2_S_4_ // FeCo_2_O_4_‐FeCo_2_S_4_	(1 m KOH) OER: 225 mV at 100 mA/cm^2^ HER: 271 mV at 100 mA/cm^2^ 1.72 V at 100 mA/cm^2^	(1 m KOH seawater) OER: 238 mV at 100 mA/cm^2^ HER: 277 mV at 100 mA/cm^2^ 1.75 V at 100 mA/cm^2^	For both HER and OER, 12 h at 100 mA/cm^2^ in 1 m KOH, 2000 cycles. In overall water splitting, 48 h	Decay by 6% within 12 h at 100 mA/cm^2^ In overall water splitting, 48 h	Constructing a FeCo_2_O_4_‐FeCo_2_S_4_ heterostructure via CoFe‐LDH transformation promotes surface reconstruction and metal oxidation, thereby enhancing corrosion resistance.	[76]
Co‐NiFeP/NIF // Co‐NiFeO_x_/NIF (NIF is nickel iron foam)	1.56 V at 10 mA/cm^2^ in 1 m KOH	1.60 V at 10 mA/cm^2^ in 1 m KOH seawater	12 h of chronoamperometry	12 h of chronoamperometry	The hierarchical, superhydrophilic, and superaerophobic nanosheet coating structure ensures full electrolyte contact and rapidly ejecting gas bubbles, helping resist chloride corrosion	[77]
1D‐Cu@Co‐CoO/Rh on copper foam // 1D‐Cu@Co‐CoO/Rh on copper foam	107.3 and 277.7 mV for HER and OER at 10 mA/cm^2^ in 1 m KOH, 1.62 V at 10 mA/cm^2^	1.7 V at 10 mA/cm^2^ in 1 m KOH natural seawater	HER, 14 h, 92.3% of J retention in 1 m KOH, OER, 98% after 11 h	Before and after 12 h of stability test, insignificant change of the current density	Anchoring metal atoms or clusters on heterostructure surfaces creates thermodynamically stable interactions, thereby improving electronic structure modulation and catalytic stability across various reactions. Embedding high‐surface‐area, conductive nanostructures with strong interfacial contact further improves reaction performance.	[78]
MoMn‐NiCoS // MoMn‐NiCoS	70 mV for HER, 247 mV for OER at 10 mA/cm^2^ 1.57 V at 10 mV/cm^2^ in 1 m KOH	73 mV for HER, 282 mV for OER at 10 mA/cm^2^ 1.62 V at 10 mV/cm^2^ in 1 m KOH	50 h ‐chronopotentiometry at 50 mA/cm^2^	50 h ‐chronopotentiometry at 50 mA/cm^2^	Heteroatom doping and phase engineering are effective strategies to enhance catalytic activity, stability, and corrosion resistance. Sulphate adsorption on transition metal hydroxides/oxyhydroxides provides electrostatic repulsion against chloride ions, improving corrosion resistance. Additionally, amorphous sulphides show superior catalytic performance and durability compared to their crystalline counterparts.	[79]
Cu_2_S‐80°C // Cu_2_S‐80°C	1.65 V at 10 mA/cm^2^ in 1 m KOH	445 mV at 10 mA/cm^2^ in natural seawater	99.23% up to 10 h in 1 m KOH	86.7% stability up to 10 h	Cu_2_S catalyst had impressive stability in nearly neutral seawater.	[80]

Across the studies compiled in Table 1, the same catalyst and device would exhibit a consistent but bounded activity decrease after switching from freshwater to seawater electrolyte. The magnitude of this activity gap depends strongly on electrolyte composition, current density, corrosion resistance of the catalyst, etc. When seawater is alkalized with KOH, the high‐performance catalysts can nearly eliminate the gap even at industrial current density. For example, Du et al. [81] reported a corrosion‐resistant NiFe layered double hydroxide electrode (CAPist‐S1) that reached 1 A/cm^2^ with only 220 mV of overpotential in seawater with 1 m KOH electrolyte. The overpotential at 1 A/cm^2^ in 0.5 m NaCl with 1 m KOH is only 20 mV lower than in alkalized natural seawater. Even though the activity of CAPist‐S1 in KOH is not reported, another catalyst CAPist‐L1 developed by the same group inspired by this work, has required 220 mV of overpotential in 1 m KOH at 1 A/cm^2^ [82]. In weakly buffered natural seawater, the activity penalty widens much more substantially comparing to alkalized seawater. For instance, Karthikeyan's group developed a bifunctional IrO_2_@MnO_2_ catalyst that required 170 and 190 mV of overpotentials for HER and OER in 1 m KOH at 10 mA/cm^2^. However, its cell voltage at a low current density of 10 mA/cm^2^ in natural seawater is 1.64 V, which is 40 mV higher than that of freshwater electrolysis [83]. The difference therefore reflects the combined influence of chloride content, buffering capacity, and electrolyte conductivity, instead of the intrinsic properties of the catalysts. These cases indicate that the freshwater to seawater gap is not an intrinsic deterioration of the catalyst but a controllable penalty that is dependent on the electrolysis environment. Catalyst design therefore works under a trade‐off: the gap between freshwater and seawater electrolysis can be suppressed by alkalization or buffering, but only by introducing reagents and system complexity that erode the cost and simplicity advantages motivating the DSE in the first place. Intrinsic catalyst activity sets the achievable performance while the electrolysis design dictates how closely a given device can approach this performance.

### 3.3 Persistent Gaps and Strategic Priorities

**FIGURE 3:**
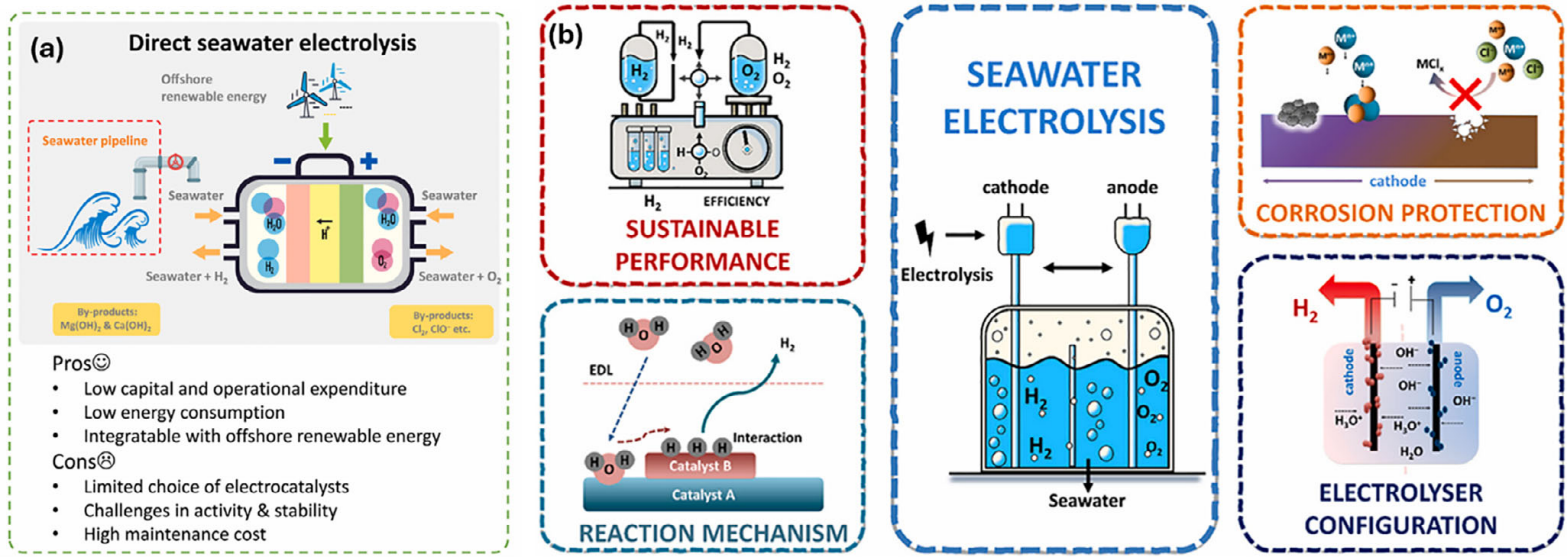
(a) Schematic illustration of direct seawater electrolysis from the aspect of technological and economic feasibility. Reproduced under terms of the CC‐BY license [20]. Copyright 2023, American Association for the Advancement of Science. (b) Schematic illustration of seawater electrolysis from the perspective of challenges, configuration, and mechanism. Reproduced with permission [88]. Copyright 2025, Elsevier.

Despite advances in catalyst and electrolyzer design, several persistent gaps continue to limit the practical deployment of seawater electrolysis technologies. To chart a viable path forward, it is essential to identify the limitations and define strategic priorities for both system‐level development and catalyst design from the perspectives of technological and economic feasibility (Figure 3a).

DSE still faces a few unsolved issues and integration barriers at the system level. The complex composition of seawater (salts, microorganisms, etc.) causes multiple failure modes: anodic ClOR, cathodic mineral scaling, and biofouling, all of which contribute to electrode corrosion and cell performance loss [20]. Advanced cell designs and materials are being explored to mitigate these challenges, but persistent gaps remain. Zero‐gap [84] and multi‐compartment [85] electrolyzer configurations with ion‐selective membranes can partially isolate electrodes from detrimental species, yet completely suppressing Cl^−^ and other ion crossover has proven difficult [7]. For example, conventional proton‐exchange membranes suffer degradation and fouling in chloride‐rich environments and under conditions that promote the formation of insoluble precipitates [56]. Another example is the state‐of‐the‐art anion exchange membranes that still face the challenge of completely blocking impurity transport. Scaling and fouling countermeasures, such as improving electrode hydrophilicity and enhancing bubble growth kinetics, show promise in laboratory tests for reducing precipitate accumulation, but translating these solutions to industrial‐scale, continuous operation is challenging [86, 87].

Overall, maintaining stable, efficient, and long‐term H_2_ production in real seawater conditions remains a major integration barrier. These fundamental hurdles in cell design, membrane functionality, and system endurance must be overcome to enable durable, scalable seawater electrolyzer deployment [56]. Addressing these persistent system‐level gaps will require both engineering advances and the development of highly efficient and resilient electrocatalysts. In the following sections, strategies from the perspectives of catalyst design, system engineering, and reaction mechanisms will be discussed, aiming to narrow these gaps (Figure 3b).

The benchmarks surveyed above expose a gap between the current status of direct seawater splitting (DSE) research and the target, sustained operation above 1 A/cm^2^ at cell voltages below ∼2 V over tens of thousands of hours [19, 62]. The diminished selectivity and accelerated degradation at industrial‐scale current densities are not independent engineering issues but can originate at the catalytic active site and its surrounding microenvironment. Addressing the system‐level issues and targets identified in this section therefore requires returning to the atomic scale to explore DSE mechanisms and overcome the bottleneck. The following section examines how catalyst composition, electronic structure, and interfacial design determine activity, selectivity, stability, and thus whether the industrial benchmarks above can be met.

## 4 Catalysts for Seawater Electrolysis

### 4.1 HER Catalysts

HER is a key half‐reaction in electrocatalytic seawater splitting for H_2_ production. HER catalysts play a vital role by providing an alternative reaction pathway with a lower activation barrier. Generally, HER catalysts can be divided into noble metal‐based and non‐noble metal‐based catalysts. This section also discusses strategies employed to enhance their catalytic activity and durability against chloride‐induced corrosion and electrode passivation.

#### 4.1.1 Noble Metal‐Based Catalysts

The division of HER catalysts into noble‐metal and non‐noble metal categories reflects a fundamental trade‐off between intrinsic activity and practical deployability that governs catalyst selection for seawater electrolysis. Noble metal‐based catalysts are regarded as benchmark materials for HER because their near‐thermoneutral hydrogen adsorption free energy places them at or near the apex of the HER activity volcano, optimally balancing the adsorption and desorption of hydrogen intermediates [89, 90]. Pt, Rh, Ru, and Os consequently possess optimal intrinsic HER activity and remain the performance standard. Compared to that in freshwater electrolysis, the merits of these catalysts are no longer defined by intrinsic activity alone in DSE. Unlike the purified electrolytes used in conventional freshwater electrolysis, natural seawater exposes the cathode to Cl^−^ that corrode and poison noble‐metal active sites, and to Mg^2+^/Ca^2+^ that precipitate once the local pH rises and physically block the surface [27]. The design of noble‐metal cathodes for seawater therefore diverges from freshwater electrolysis in two aspects. First, activity optimization is supplemented by anti‐corrosion and anti‐fouling engineering, such as protective overlayers or interfacial fields that electrostatically repel Cl^−^ and suppress precipitate formation near the active site [91]. Second, because the high cost and scarcity of noble metals are aggravated at the industrial DSE conditions, contemporary design emphasizes maximizing noble‐metal utilization through atomic dispersion, single‐atom configuration, and alloying, retaining benchmark activity at a reduced loading [92].

Pt is widely recognized as the most efficient HER catalyst, primarily because of its near‐zero Gibbs free energy for hydrogen adsorption (Δ*G*_H*_), which ensures an optimal balance between the adsorption and desorption of hydrogen intermediates (H*). Pt doping can further enhance catalytic performance by generating electron‐rich active sites and promoting hydrogen spillover. These synergistic effects accelerate water dissociation and boost HER kinetics, resulting in superior activity and stability. For example, a Pt‐doped Ni_2_P catalyst with phosphorus vacancies (Pt@Ni_2_P_v_) exhibits outstanding HER activity under alkaline seawater conditions [93]. The presence of P vacancies promotes charge transfer to Pt nanoclusters, while Pt nanocluster doping improves the H_2_ spillover to adjacent Ni sites. This synergistic structural modification accelerates HER kinetics by generating electron‐rich Pt sites, which promote water dissociation to H* and facilitate subsequent H_2_ desorption. Therefore, Pt@Ni_2_P_v_ demonstrates excellent activity (89 mV at 1000 mA/cm^2^). Pt nanoclusters are also loaded on NiFeCo‐P (Pt@NiFeCo‐P) to further improve HER activity and stability [94]. Under alkaline seawater conditions, Pt@NiFeCo‐P exhibits an ultralow overpotential of 19.7 mV at 10 mA/cm^2^ and maintains exceptional stability for 1000 h at 1000 mA/cm^2^. This outstanding performance arises from the strong metal‐support interaction between the Pt nanoclusters and the NiFeCo‐P substrate, which facilitates rapid electron transfer from NiFeCo‐P to the Pt sites. Consequently, this interaction results in high coverage of adsorbed H* and accelerates water dissociation on Co sites, collectively boosting the HER kinetics.

Apart from Pt‐based catalysts, other noble metal‐based catalysts are also being explored. Recently, Os has attracted much attention as an HER catalyst due to its distinctive electronic structure and intrinsic HER activity. For example, boron‐doped osmium (B‐Os) is synthesized by a microwave quasi‐solid approach. B doping induces electronic enrichment at Os sites and the formation of negative charge centers, which promote outstanding activity (7 mV at 10 mA/cm^2^) and stability (100 h at 500 mA/cm^2^) in alkaline seawater [95]. B‐Os also maintains remarkable stability for 400 h at 1000 mA/cm^2^ in the anion exchange membrane (AEM) system. In addition, Rh doping can also affect the electronic structure of catalysts to improve HER activity. Gao et al. synthesized Rh nanocrystals doped into porous graphdiyne (Rh/GDY) by a wet‐chemical reduction reaction [96]. The overpotential of Rh/GDY is only 65 mV at 1000 mA/cm^2^ under alkaline seawater environments.

#### 4.1.2 Non‐Noble Metal‐Based Catalysts

Noble metal‐based catalysts possess excellent activity for HER, while the limitations such as high cost and scarcity restrict their industrial application. This economic constraint is the primary reason for the development of the earth‐abundant non‐noble metal compounds, including transition‐metal phosphides, sulfides, nitrides, oxides/hydroxides and their alloys and heterostructures [97, 98]. The non‐noble metal catalysts not only have lower cost, but also comparable activity with noble metals. For seawater specifically, the chloride corrosion and cation‐precipitation that occurred on the cathode put a high request for intrinsic chemical robustness. Several non‐noble compound classes show that their metallic conductivity offsets the sluggish HER kinetics in the unbuffered seawater, while their tunable compositions simplify incorporating corrosion resistance engineering [99, 100]. Compared to freshwater systems, catalysts in DSE are deliberately tailored for Cl^−^ tolerance and anti‐precipitation behaviors, rather than optimizing the hydrogen evolution steps alone [101].

Molybdenum (Mo)‐based catalysts are regarded as highly active materials for HER owing to their tunable electronic structure and compatibility with reaction intermediates. Molybdenum nitride (Mo_2_N) demonstrates great activity (311 mV at 10 mA/cm^2^) and stability for 1000 h at 100 mA/cm^2^ in natural seawater [102]. Similarly, Ni and MoN nanoparticles on the amorphous MoN nanorods form a heterogeneous Ni‐MoN catalyst [103]. Ni‐MoN possesses a hierarchical nanorod‐nanoparticle structure, large surface area, and multidimensional boundaries/defects, which provide numerous active sites. The combination of Ni and MoN regulates the electron redistribution and enhances water‐dissociation kinetics at Mo sites. Ni‐MoN thus requires a low overpotential 66 mV at 100 mA/cm^2^ in alkaline seawater. In addition, a yttrium‐doped NiMo/MoO_2_ (Y‐NiMo/MoO_2‐x_) heterogeneous electrocatalyst demonstrates remarkable HER activity [104]. It only requires 220 mV to achieve 2000 mA/cm^2^ and maintains outstanding stability for 2000 h at 2000 mA/cm^2^ in alkaline seawater. Due to Y doped into NiMo/MoO_2_, lattice expansion is induced, and the *d*‐band center of the NiMo alloy is optimized, thereby promoting H_2_O dissociation and H* desorption. Y doping also increases oxygen vacancies in MoO_2‐x_, which accelerates the charge kinetics and detachment of *OH from the active sites.

Other transition metal‐based materials (e.g., Fe, Co, Ni), including phosphides [105, 106] and sulfides [107], are also investigated as HER catalysts. For example, CoP grown in situ on rGO@Ti forms a catalyst (CoP/rGO@Ti), that demonstrates excellent HER activity (210 mV at 200 mA/cm^2^) and stability in alkaline seawater. This is attributed to a repulsive effect between CoP and Cl^−^. Besides, Li et al. constructed a NiCoS heterostructure with nitrogen‐doped carbon shell encapsulation (CN@NiCoS) electrocatalyst [107]. Experimental and computational methods reveal that NiCoS induces S migration by creating S vacancies at the Ni_3_S_2_‐Co_9_S_8_ heterointerface. The migrated S then combines with the CN shell to form a strong C─S bond, which protects S dissolution in alkaline conditions. Meanwhile, the former C─S bond and S vacancies shift the *d*‐band center toward the Fermi level, which promotes the HER activity. These modifications result in great activity (8 mV at 10 mA/cm^2^) and outstanding stability for 1000 h at 1000 mA/cm^2^.

#### 4.1.3 Design Strategies for Advanced Cathodes

Precipitates and chlorine corrosion will occur during seawater splitting because seawater contains many ions (Cl^−^, Mg^2+^, and Ca^2+^). Therefore, the intrinsic activity of HER is merely one aspect of the cathode design in seawater electrolysis. To address these problems, other strategies are employed to promote the activity and stability of HER catalysts, including self‐cleaning and local microenvironment regulation. It is noteworthy that the strategies discussed below can all be summarized to one single mechanistic origin, which is that the cathodic HER intrinsically alkalizes the catalyst–electrolyte interface. Whether the reaction proceeds through direct proton reduction or water dissociation, every evolved H_2_ molecule leaves behind two hydroxide ions [108].

**FIGURE 4:**
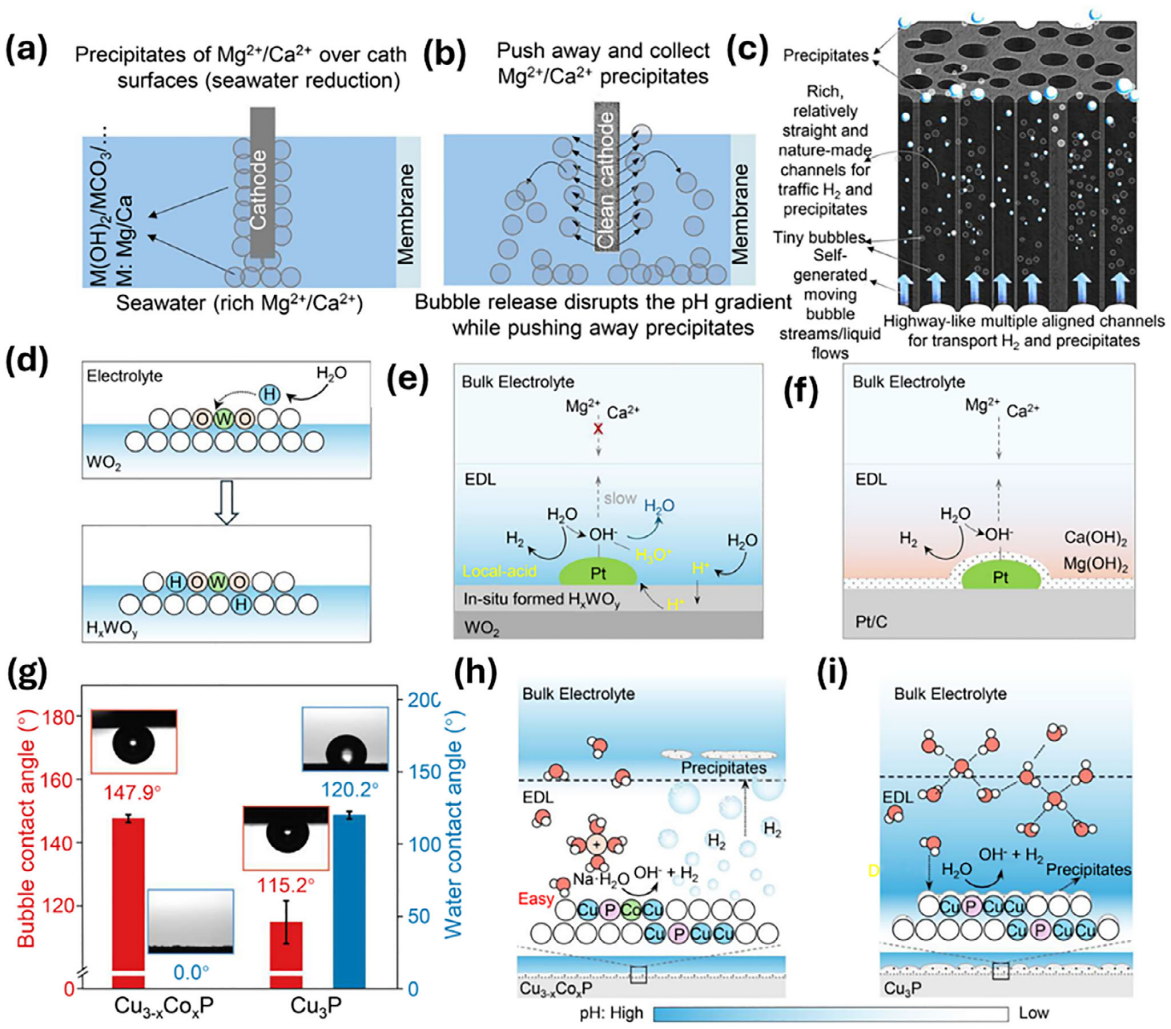
(a,b) Mechanisms of precipitate formation on different cathodes. (c) Advantages of 3D architecture cathode in repelling precipitation. Reproduced with permission [109]. Copyright 2024, Elsevier. (d) Mechanism of in situ formation of HxWOy. (e,f) Schematic for HER mechanism on Pt/WO_2_ and Pt/C. Reproduced with permission [110]. Copyright 2024, American Chemical Society. (g) Static contact angles of bubble (red) and water droplet in air (blue) for catalysts. (h,i) Schematic illustrations for HER mechanism in natural seawater on Cu_3‐x_Co_x_P and Cu_3_P. Reproduced with permission [112]. Copyright 2025, American Chemical Society.

The formation of hydroxide precipitates at the cathode (Figure 4a) is a major problem during the HER process, because they deposit on the catalyst surface and decrease catalytic activity. To solve this problem, a self‐cleaning cathode (Figure 4b) has been designed [109]. A 3D Pt/C consisting of rich channels with low tortuosity (Figure 4c) can generate flows to repel precipitates. This phenomenon is attributed to the fact that the precipitates formed at the cathode are readily influenced by air/liquid flow. The regular channels form tiny bubbles, which can carry away the precipitates to achieve self‐cleaning (Figure 4c). Similarly, Liang et al. also investigated a microscopic bubble/precipitate traffic system by establishing a honeycomb‐type 3D cathode (NiCoP decorated pinewood‐derived carbon, NCP/PC) for anti‐precipitate seawater splitting [86]. The honeycomb‐type structure of NCP/PC uniformly releases tiny H_2_ bubbles to constantly take away almost every corner Mg^2+^/Ca^2+^ precipitate. Therefore, NCP/PC exhibits great activity (550 mV at 500 mA/cm^2^) and outstanding stability of the two‐electrode system (150 h at 500 mA/cm^2^) in natural seawater. Self‐cleaning is inherently a remedial strategy. The precipitate is not avoided from forming but is cleared afterward. Its efficacy stems from electrode architecture rather than intrinsic catalyst chemistry. Rather than a limitation, this feature is advantageous, indicating this approach, with channel geometry, honeycomb porosity, and the resulting bubble traffic governing performance, is robust, scalable, and potentially transferable across catalyst compositions.

Local microenvironment regulation is another strategy that can suppress precipitation and reduce chlorine corrosion. By establishing a local acid‐like environment on the catalyst surface, the formation of precipitation can be avoided. As shown in Figure 4d, WO_2_ in Pt/WO_2_ acts as active sites for adsorption and cleavage of H_2_O, and hydrogen then inserts into an interstitial lattice site of WO_2_ to in situ form H*_x_* WO*_y_* during the HER process [110]. The H^+^ in H*_x_* WO*_y_* transfers to Pt and concentrates in the electric double layer (EDL) at the Pt surface. Numerous H_3_O^+^ establish a local acid‐like environment that promotes HER activity and neutralizes concentrated OH^−^ derived from HER in natural seawater (Figure 4e). This process reduces the microenvironmental pH of Pt/WO_2_ to hinder precipitation. Additionally, the electrostatic repulsion between OH^−^ and Cl^−^ enhances the chlorine corrosion resistance. In contrast, for Pt/C, H_2_O dissociation initiates on the Pt surface, generating H^+^ and OH^−^, and then H^+^ couples for HER. The OH^−^ aggregates and forms precipitation, resulting in HER activity decay (Figure 4f). Equally, amorphous NiMoB also creates a local acid‐like environment on the surface due to in situ generation of H_3_O^+^ during the HER process [111]. This results in excellent HER activity (39.4 mV at 10 mA/cm^2^) in natural seawater. The strain‐engineered strategy can modify the local pH gradient and hinder precipitation formation. Cu_3‐x_Co_x_P with lattice compressive strain modifies interfacial H_2_O behavior and enhances surface hydrophilicity (Figure 4g), thereby promoting H_2_O dissociation and bubble release [112]. The mechanism of HER on Cu_3‐x_Co_x_P and Cu_3_P is shown in Figure 4h,i. For Cu_3‐x_Co_x_P, the introduction of Co shortens the Cu–Cu distance and causes lattice compressive strain, accelerating the adsorption of Na^+^ on the catalyst surface during the HER process in natural seawater. The concentration of Na^+^ increases Na·H_2_O coverage on the surface to improve H_2_O dissociation. Meanwhile, H_2_ bubbles release rapidly due to the superhydrophilic and aerophobic properties of Cu_3‐x_Co_x_P, thereby modifying the local pH gradient on the catalyst surface and hindering precipitation formation. However, H_2_O dissociation on the Cu_3_P surface is difficult owing to the strong hydrogen‐bonded H_2_O. After H_2_O dissociation, OH^−^ aggregates on the Cu_3_P surface and then forms precipitation, resulting in HER activity decay. A concise overview of recent HER catalysts and their corresponding seawater performance is presented in Table 2. These local environment regulation strategies all create a local acid‐like environment on the catalyst surface to inherently avoid the precipitation of hydroxides. However, there are differences in their mechanisms. Pt/WO_2_ [110] and NiMoB [111] act at the alkalization step, supplying the interfacial protons (H_3_O^+^) that neutralize the OH^−^ released by HER and thereby cap the local pH rise at its source. Cu_3‐x_Co_x_P [112] deploys wettability engineering and acts one step earlier on the generation and transport of OH^−^ by tuning H_2_O dissociation kinetics, promoting Na^+^ enrichment, and accelerating bubble departure through a superhydrophilic/aerophobic surface.

**TABLE 2:** Performance comparison of recent HER catalysts in seawater splitting.

Catalyst	Electrolyte	Overpotential (mV@*η*_10_)	Stability	Refs.
Pt@Ni_2_P_v_	1 m KOH seawater	*η*_100 _= 37.2	1200 h@1000 mA/cm^2^	[93]
Pt@NiFeCo‐P	1 m NaOH+0.5 m NaCl	19.7	1000 h@1000 mA/cm^2^	[94]
Pt/WO_2_	Natural seawater	290	500 h@100 mA/cm^2^	[110]
Pt‐SA/NiV‐LDH	1 m KOH seawater	10	500 h@500 mA/cm^2^	[113]
B‐Os	1 m KOH seawater	7	100 h@500 mA/cm^2^	[95]
FeCoNiMnRu	1 m KOH+0.5 M NaCl	36	12 h@20 mA/cm^2^	[114]
RuMo/Cu_2_O@C	1 m KOH seawater	24	300 h@500 mA/cm^2^	[115]
NiRh‐MOF	1 m KOH seawater	49	60 h@10 mA/cm^2^	[116]
Rh/GDY	1 m KOH+0.5 m NaCl	η_100_ 28	30 h@1000 mA/cm^2^	[96]
Ni‐MoN	1 m KOH seawater	24	100 h@500 mA/cm^2^	[103]
Y‐NiMo/MoO_2‐x_	1 m KOH seawater	*η*_100 _= 56	2000 h@2000 mA/cm^2^	[104]
CoP/rGO@Ti	1 m KOH + 0.6 m NaCl	110	60 h@10 mA/cm^2^	[106]
NCP/PC	1 m KOH seawater	*η*_100 _= 92	1000 h@1000 mA/cm^2^	[86]
NiMoB	1 m KOH +0 .5 m NaCl	23	100 h@1000 mA/cm^2^	[111]
Cu_3‐x_Co_x_P	Natural seawater	219	1000 h@100 mA/cm^2^	[112]

The design strategies for DSE cathodes, though they vary in mechanism, share a common objective, which is to regulate the interfacial pH and ion environment. For DSE, this objective is as important as the cathodic HER activity. Table 2 shows that most of these demonstrations operate at high current densities that are close to industrial seawater electrolysis; the intensified OH^−^ production proves the viability of these strategies.

### 4.2 OER Catalysts

OER is a complex four‐electron transfer and kinetically sluggish process, which also leads to high overpotential. Therefore, OER catalysts are crucial to improve the efficiency of seawater electrolysis. Currently, many materials, such as HEAs, oxides, and metal–organic frameworks (MOFs), are explored as OER catalysts. For a simpler classification, here the OER catalysts are divided into two categories: noble metal‐based and non‐noble metal‐based catalysts. The strategies for enhancing the selectivity and stability of the anode are also discussed.

#### 4.2.1 Noble Metal‐Based Catalysts

Noble metal‐based catalysts are promising for seawater splitting because they have high electrical conductivity and catalytic activity. Ir and Ru possess tunable electronic structure and suitable intermediate adsorption energies for OER, which results in excellent OER performance. Meanwhile, IrO_2_ and RuO_2_ are benchmarks for OER. Therefore, Ir‐/Ru‐based catalysts are regarded as highly active materials for OER.

Ir loaded on NiOOH‐Se (Ir/NiOOH‐Se) presents great OER performance because of the interaction between Ir and Cl^−^ [117]. This interaction constructs a Cl─Ir─O─Ni electron‐withdrawing chain from Ni to Cl, which enhances Ni─O covalency, thereby activating lattice oxygen around Ni sites. Therefore, Ir/NiOOH‐Se desires low overpotential of 313 mV to achieve 500 mA/cm^2^ and shows good stability for 500 h at 500 mA/cm^2^ in alkaline seawater. In addition, single‐atom Ir deposited onto CoFe‐layer double hydroxide (Ir/CoFe‐LDH) modifies Cl^−^ adsorption and the electronic structure of the Ir active center, establishing a unique Ir‐OH/Cl coordination during alkaline seawater oxidation [118]. Ir‐OH/Cl coordination promotes the adsorption of *OOH intermediates and reduces the activation energy barrier. This results in Ir/CoFe‐LDH demonstrating excellent activity (236 mV at 10 mA/cm^2^) and stability (1000 h at 800 mA/cm^2^). Graphdiyne grown on IrCuO_x_ (GDY/IrCuO_x_) exhibits great OER activity due to strong heterointerface strains between GDY and IrCuO_x_ [119]. To achieve 1000 mA/cm^2^, GDY/IrCuO_x_ requires only 283 mV in alkaline seawater. Furthermore, Ru_0.1M_n_0.9_O_x_ is investigated as an OER catalyst in acidic seawater [120]. During the OER process, *Cl adsorbed on Ru sites can shift the OER active center Mn on Ru_0.1_Mn_0.9_O_x_, which suppresses the dissolution of metal sites and consumption of lattice oxygen. This behavior enhances the selectivity and stability (1200 h at 10 mA/cm^2^) during acidic OER.

Except for Ir‐/Ru‐based catalysts, other noble metals such as Os and Ag are also investigated as OER catalysts in seawater splitting. For instance, Os‐Ni_4_Mo/MoO_2_ micropillar arrays with metal–support interaction present excellent OER activity in natural seawater [121]. It only requires 336 mV overpotential to achieve 500 mA/cm^2^ current density. This great performance is attributed to the micropillar structure enhancing electron and mass transfer and extending catalytic reaction steps. The metal–support interaction between Os and Ni_4_Mo/MoO_2_ optimizes the surface electronic structure and reduces the reaction barrier. Additionally, Ag can also improve the OER performance by interacting with Cl^−^. Ag nanoparticles loaded on NiFe‐LDH (NiFe‐LDH@Ag) exhibit remarkable stability for 2500 h at 400 mA/cm^2^ [122]. This is attributed to free Cl^−^ reacting with oxidized Ag nanoparticles to form stable AgCl species, thereby generating a Cl^−^‐free layer near the catalyst surface.

#### 4.2.2 Non‐Noble Metal‐Based Catalysts

Transition metal‐based catalysts have attracted much attention because of their low cost, abundant availability, and promising catalytic performance. Their competitive activity, stability, and tunability make them suitable options for OER.

Fe, Co, and Ni are the most common transition metal elements used as OER catalysts. For example, CoFe‐based anode performance is modified by docking PW_12_‐polyoxometalate (PW_12_‐POM) [123]. PW_12_‐POM anchors onto Fe sites of CoFe‐LDH anode, which modulates the electronic structure of adjacent Co active sites and modifies the adsorption of Cl^−^/OH^−^ during alkaline seawater oxidation. The overpotential of PW_12_‐CoFe‐LDH is only 368 mV at 1000 mA/cm^2^. Impressively, PW_12_‐CoFe‐LDH maintains 1300 h stability at 1000 mA/cm^2^. Similarly, NiFe‐LDH is also a common catalyst investigated for seawater oxidation. Recently, a corrosion‐resistant NiFe‐LDH (CAPist‐S1) was reported [124]. This catalyst requires only 220 mV to achieve 1000 mA/cm^2^ and maintains 9000 h stability at 1000 mA/cm^2^ in alkaline seawater. In an AEM system, CAPist‐S1 demonstrates remarkable stability for 700 h at 1000 mA/cm^2^. Due to the generation of a dense NiFe‐LDH interlayer between NiFe‐LDH nanosheets and the metal substrate, a dynamic equilibrium is established between Fe dissolution and redeposition over the in situ formed FeOOH, thereby maintaining OER activity. In addition, the change of metal spin state can also promote the OER performance of catalysts. An asymmetric Fe‐doped NiPS_3_ (Fe/NiPS_3_) demonstrates a low overpotential of 264 mV at 10 mA/cm^2^ in alkaline seawater [125]. In an AEM system, Pt/C||Fe/NiPS_3_ requires a low cell voltage around 1.52 V at 10 mA/cm^2^ and keeps superior stability for 1000 h under alkaline seawater conditions. Experimental investigations reveal that the presence of Ni^3+^ induces the formation of medium spin (MS) Fe^3+^ to constitute a spin‐selective electron transfer channel in the Fe─O─Ni bridge. As a result, Fe/NiPS_3_ presents excellent OER performance. High spin (HS)‐Fe_0.17_Co_0.83_Se_2_ with high spin Co sites is also researched as an OER catalyst [126]. Due to the selective adsorption of Cl^−^ on the high‐spin Co sites in seawater, catalyst reconstruction and fast generation of high‐valence Co are induced, which results in the formation of additional Co active sites to promote electrolyte transport. Therefore, HS‐Fe_0.17_Co_0.83_Se_2_ exhibits excellent activity (377 mV at 1000 mA/cm^2^) and stability for 250 h at 1000 mA/cm^2^ in alkaline seawater.

Other transition metals such as Mo and Mn are also explored for OER catalysts. An amorphous MoO_3_ layer constructed on the beaded‐like CoO interface on the carbon cloth (MoO_3_@CoO/CC) exhibits great activity (650 mV at 800 mA cm^−2^) and long‐term stability over 1000 h at 600 mA cm^−2^ [127]. During the OER process, MoO_3_@CoO/CC undergoes self‐reconstruction and in situ forms CoMo‐LDH, which enhances the OER performance. Meanwhile, HEAs are regarded as potential catalysts due to their thermodynamic stability, excellent corrosion resistance, and tunability [128]. A Janus‐architected heterophase catalyst of HEA(NiFeCoCuMo)‐Mo_2_C is investigated [129]. The heterophase architecture supports dynamic interface reconstruction during the OER process and in situ forms MoO_4_2^−^ on the surface of the HEA phase. In situ formed MoO_4_^2−^on Mo_2_C generates a stable anionic protective layer, which repels Cl^−^ due to electrostatic repulsion. These features result in remarkable OER activity of 333 mV at 500 mA/cm^2^ and great stability for 250 h at 100 mA/cm^2^. In addition, MOFs have attracted extensive attention owing to their high surface area, tunable porosity, and modifiable active sites [130]. FeMn‐MOF is reported as a catalyst for alkaline seawater oxidation [131]. The metal centers in FeMn‐MOF present strong and prior coordination with OH^−^, which improves OER activity and hinders chlorine corrosion. Therefore, FeMn‐MOF requires 464 mV at 1000 mA/cm^2^ and maintains great stability for 500 h at 100 mA/cm^2^. In an industrial‐scale AEM stack, it just needs 1.8 V to achieve 20 A, which has potential for industrial application.

#### 4.2.3 Design Strategies for Advanced Anodes

During seawater oxidation, the selectivity and corrosion resistance need to be considered when designing anodes. This is because chlorine oxidation is a competitive reaction for OER. Therefore, apart from modifying the intrinsic activity of the OER catalysts, other strategies including Lewis acid regulation, chlorine mediation, anion passivation, and corrosion engineering have also been taken into consideration to address these challenges. The two defining challenges of the seawater anode: the loss of OER selectivity to the competing ClER and the accelerated corrosion of active sites, share a common origin in the anionic environment that develops at a dynamically reconstructing surface. Under anodic potential, OH^−^ (in alkaline and neutral DSE) and Cl^−^ are simultaneously driven toward the electrode, and the balance between them at the interface governs which reaction proceeds and leads to the status of the active sites. Seawater anode design therefore reduces to a single objective, which is regulating the interfacial OH^−^/Cl^−^ balance while stabilizing the reconstructed surface [11].

**FIGURE 5:**
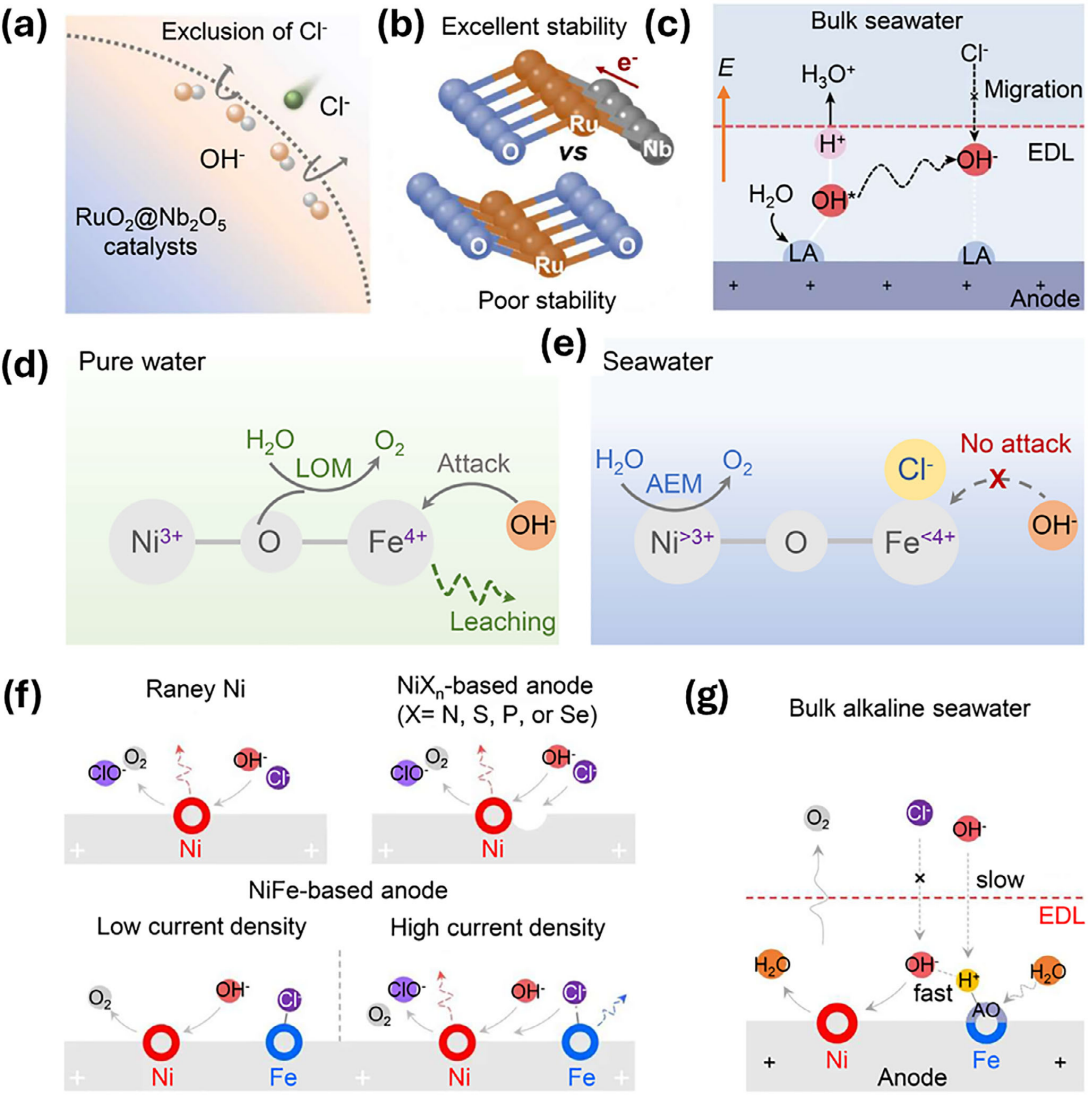
(a,b) Mechanism diagrams of RuO2@Nb2O5 for OER in neutral seawater. Reproduced with permission [132]. Copyright 2025, Elsevier. (c) Schematic illustration of a local alkaline environment formed on a Lewis acid‐modified anode, which promotes OER and hinders Cl‐ oxidation. Reproduced with permission [22]. Copyright 2023, Springer Nature. (d,e) Schemes for structure evolution and OER mechanism transfer in alkaline pure water and alkaline seawater. Reproduced under terms of the CC‐BY license [21]. Copyright 2023, John Wiley and Sons. (f) Schematic images for ions’ behaviors under different conditions. (g) Schematic diagram for ions’ behaviors upon NiFe LDH‐[A] anode surface. Reproduced under terms of the CC‐BY‐NC‐ND 4.0 license [72]. Copyright 2024, Springer Nature.

Lewis acid regulation is an effective strategy to improve catalytic selectivity and suppress chlorine corrosion. Lewis acid layers such as Nb_2_O_5_ and Cr_2_O_3_ are introduced on catalysts to dynamically dissociate H_2_O and capture OH^−^. In situ formed local alkaline environment promotes OER activity and avoids chlorine attack. For example, RuO_2_@Nb_2_O_5_ presents high selectivity (98.5% at 500 mA/cm^2^) and stability (over 100 h at 100 mA/cm^2^) for OER in neutral seawater [132]. This performance is attributed to the Lewis acid supports accelerating OH^−^ adsorption. The generated OH^−^ layer repels Cl^−^ and decreases the binding affinity between Ru active sites and Cl^−^ (Figure 5a). Nb_2_O_5_ also serves as the electron donor for RuO_2_ (Figure 5b), which avoids high‐valent Ru dissolution and enhances stability. Similarly, a Lewis acid‐modified catalyst (Cr_2_O_3_–CoO_x_) is reported [22]. In neutral seawater, H_2_O splitting tends to occur on Cr_2_O_3_ due to its low energy barrier for H_2_O dissociation. Then, large amounts of *OH generated from H_2_O dissociation cover the Cr_2_O_3_–CoO_x_ surface, leading to a gradual weakening of *OH adsorption on Cr_2_O_3_ and accelerating *OH desorption to form OH^−^ in the electrical double layer (Figure 5c). Therefore, the in situ formed local alkaline environment promotes OER activity and repels Cl^−^ by electrostatic repulsion. Mechanistically, Lewis acid regulation is the anodic counterpart to local‐acid engineering at the cathode, which was discussed in Section 4.1.3. Instead of simply neutralizing interfacial OH^−^, the oxide captures and accumulates hydroxide ions, creating a localized alkaline microenvironment that is decoupled from the bulk electrolyte, thereby sustaining efficient HER in neutral seawater. Its effectiveness arises from three coupled effects acting on the same interface: OH^−^ enrichment that favors OER over ClER, OH^−^ layer repelling Cl^−^, and charge donation from the support that suppresses over‐oxidation and dissolution of the metallic electrode [133]. Lewis acid regulation simultaneously improves reaction selectivity and corrosion resistance, with its benefits being most pronounced in neutral direct seawater electrolysis.

A chlorine‐mediated strategy can stabilize the catalyst structures and prevent chlorine corrosion. A traditional NiFe‐based catalyst is modified by small organic molecules (SOMs) featuring an aromatic carbon ring with para‐positioned carbonyl groups [134]. SOMs preferentially adsorb Cl^−^ to hinder the interaction of metal active sites and Cl^−^. SOM‐Cl acts as an electron buffering group to reduce the dissolution of Fe sites during OER, which promotes anode stability [21]. In situ characterizations reveal that Cl^−^ is adsorbed on Fe sites, which prevents Fe leaching and creates more Ni active centers. In alkaline pure water, the valences of Fe and Ni increase to Ni^3+^ and Fe^4+^ via a lattice oxygen mechanism during the OER process (Figure 5d). Fe^4+^ becomes a relatively hard acid and is then attacked by OH^−^, which causes Fe leaching and poor durability. In contrast, Fe is preserved from OH^−^ attack due to Cl^−^ adsorption on the Fe site in alkaline seawater (Figure 5e). This interaction hinders Fe leaching and increases Ni valence. The high‐valent Ni becomes an OER active center, which stabilizes lattice oxygen. As a result, NiFe‐LDH demonstrates remarkable durability for 100 h at 200 mA/cm^2^ in alkaline seawater. Unlike conventional strategies that aim to repel Cl^−^, chlorine mediation deliberately directs Cl^−^ adsorption to specific active sites or buffering groups, fundamentally inverting the catalyst design paradigm. The NiFe‐LDH case shows that Cl^−^ serves as a protective layer and fundamentally controls the catalyst's redox evolution [21]. The approach is not without limitations, as the elevated Cl^−^ flux under high anodic current densities can destabilize the Cl^−^‐covered surface, leading to surface degradation.

Anion passivation is a promising strategy to protect the anode from chlorine corrosion. By introducing CO_3_^2−^ into CoFe‐LDH interlayers and surface anchoring of graphene quantum dots (GQDs), the adsorption of Cl^−^ on the catalyst (CoFe‐C_i_@GQDs) is prevented [135]. The inserted CO_3_^2−^ generates a strong electrostatic interaction with the positively charged host layer, which enhances the structural stability of CoFe‐C_i_@GQDs and repels Cl^−^. GQDs modification also repels Cl^−^ to improve the corrosion resistance of CoFe‐C_i_@GQDs. Simultaneously, GQDs modification can stabilize high‐energy intermediate states of surface‐active sites and accelerate H_2_O dissociation. As a result, CoFe‐C_i_@GQDs requires 255 mV to achieve 100 mA/cm^2^ and keeps superior stability for 2800 h at 1250 mA/cm^2^ under alkaline seawater conditions. In addition, anion passivation‐modified catalyst surfaces can also protect anodes from chlorine corrosion. Phytic acid (PA) used as a molecular “armor” modifies NiMoO_4_@NiSe_2_ to form a high‐performance catalyst (NiMoO_4_@NiSe_2_‐PA) [136]. Experimental and calculational studies reveal that surface modification with PO_4_^3−^ promotes surface reconstruction to generate NiOOH active sites and optimizes adsorption–desorption dynamics during OER. Simultaneously, the PO_4_3^−^ layer hinders Cl^−^ adsorption on active sites, thereby enhancing corrosion resistance. Therefore, NiMoO_4_@NiSe_2_‐PA presents outstanding activity (208 mV at 100 mA/cm^2^) and stability over 1500 h at 100–600 mA/cm^2^. The basicity of the intercalated anions in NiFe LDH regulates OER activity and corrosion resistance [72]. As shown in Figure 5f, Raney Ni and NiX_n_‐based (*X* = N, S, P, or Se) anodes exhibit poor durability. For the NiFe‐based anode, the Ni site acts as an active center, and the Fe site adsorbs Cl^−^ at low current density, thereby maintaining activity and stability. However, selective adsorption of Cl^−^ on Fe sites fails to resist the greater Cl^−^ attack at high current density, which causes severe anode corrosion. To solve this problem, highly basic anions (i.e., PO_4_^3−^) anchor Fe sites and facilitate proton transfer to improve activity and durability (Figure 5g). A high concentration of OH^−^ suppresses Cl^−^ adsorption, leading to high selectivity and great corrosion resistance. As a result, NiFe LDH‐[PO_4_3^−^] demonstrates excellent durability for 1000 h at 1000 mA/cm^2^ in alkaline seawater. Anion passivation is the most multifunctional of these three strategies, repulsing Cl^−^ by a fixed negative layer, structurally reinforcing the host, enriching interfacial OH^−^, and modulating intermediate adsorption. Compared to chlorine mediation, it is able to resist Cl^−^ at higher current density because the highly basic anions can pin the active sites and raise local OH^−^ concentration enough to outcompete the intensified Cl^−^ flux [72].

A summary of the recent OER catalysts reported for seawater electrolysis, together with their performance, is given in Table 3.

**TABLE 3:** Performance comparison of recent OER catalysts in seawater splitting.

Catalyst	Electrolyte	Overpotential (mV@*η*_100_)	Stability	Refs.
Ir/NiOOH‐Se	1 m NaOH seawater	390	500 h@500 mA/cm^2^	[117]
Ir/CoFe‐LDH	6 m NaOH + 2.8 m NaCl	*η*_10 _= 202	1000 h@800 mA/cm^2^	[118]
Os‐Ni_4_Mo/MoO_2_	1 m KOH seawater	*η*_500 _= 113	2500 h@500 mA/cm^2^	[121]
NiFe‐LDH@Ag	1 m NaOH seawater	*η*_100 _= 370	2500 h@400 mA/cm^2^	[122]
Ru_0.1_Mn_0.9_O_x_	0.01 m HClO_4_ seawater	*η*_10 _= 210	1200 h@10 mA/cm^2^	[120]
RuO_2_@Nb_2_O_5_	Natural seawater	318	110 h@100 mA/cm^2^	[132]
PW_12_‐CoFe‐LDH	1 m KOH seawater	259	1300 h@1000 mA/cm^2^	[123]
CAPist‐S1	1 m KOH seawater	*η*_1000 _= 220	9000 h@1000 mA/cm^2^	[124]
Fe/NiPS_3_	1 m KOH seawater	*η*_10 _= 264	200 h@50 mA/cm^2^	[125]
HS‐Fe_0.17_Co_0.83_Se_2_	1 m KOH seawater	*η*_1000 _= 377	250 h@1000 mA/cm^2^	[126]
MoO_3_@CoO/CC	1 m KOH + 0.5 m NaCl	389	100 h@100 mA/cm^2^	[127]
HEA‐Mo_2_C	1 m KOH seawater	276	250 h@100 mA/cm^2^	[129]
FeMn‐MOF	Natural seawater	299	100 h@100 mA/cm^2^	[131]
NiMoO_4_@NiSe_2_‐PA	1 m KOH seawater	*η*_10 _= 96	250 h@100 mA/cm^2^	[136]

### 4.3 Bifunctional Catalysts

To facilitate the broader usage of electrolyzers in industrial applications, it is crucial to address the challenges of electrolyzers, such as high costs, stability concerns, and complex design. Developing efficient and stable bifunctional electrocatalysts that support both OER and HER concurrently is considered a promising solution [137].

Before surveying the specific materials, it is useful to clarify how bifunctional performance is evaluated, as the relevant criteria differ from those used for HER or OER alone. Three metrics are most commonly reported. The first is the overall cell voltage required to reach a target current density (typically 100, or 500–1000 mA/cm^2^) in a two‐electrode configuration with the reported catalyst serving as both anode and cathode. The second is the sum of the individual HER and OER overpotentials measured separately in a three‐electrode cell, which presents the intrinsic catalytic activity for the two half reactions separately. The third is stability under continuous operation, which is critical for DSE. The robustness of electrodes is essential for long‐term durability in DSE, especially for bifunctional catalysts, because the design needs to address the distinct challenges at both anode and cathode, including pH gradients, chloride‐induced degradation, precipitation blockage, etc. [138].

#### 4.3.1 Noble Metal‐Based Catalysts

As mentioned above, noble metals (e.g., Pt, Ru, Ir) are commonly utilized as water‐splitting catalysts. However, to the best of our knowledge, none of them are adequate as bifunctional seawater splitting catalysts. This is due to the singular behavior of these materials that makes them favorable for only one of the reactions. To overcome this limitation, one strategy is to combine two noble metals into a single composite, allowing each component to drive one half‐reaction. Following this design principle, Peng et al. reported a fully noble metal composite Pt@RuO_x_ for seawater splitting, reporting only 20 mV for HER, 157 mV for OER, and 1.44 V cell voltage at 10 mA/cm^2^ in acidic seawater electrolyte [139]. The introduction of Pt could increase oxygen vacancies, and the synergistic effect between Pt and Ru enhanced the catalytic performance significantly. It is possible that the lack of reported materials in this category is due to the inherent instability of these materials, particularly Ru in seawater conditions, whereby the high overpotentials can result in the onset of chlorine oxidation and corrosion of the catalyst material [140, 141]. Therefore, alternatives in material design that eliminate the use of noble metals or incorporate them in minimum quantities are highly desirable.

One pathway for minimizing quantities of noble metals and maximizing the utilization efficiency is to develop single‐atom catalysts, in which isolated noble‐metal atoms are anchored on the host. A few single‐atom catalysts that can be deployed in seawater electrolysis have been reported. For example, Feng's group [142] presented a Ru Sas‐MoO_3‐x_/NF that requires only 209 mV for OER, 36 mV for HER, and 1.487 V cell voltage to reach 10 mA/cm^2^ in alkaline media. While in alkaline seawater electrolysis, only a low cell voltage of 1.759 V is required to reach 100 mA/cm^2^. Wen et al. [44] developed a large‐scale spinel Ni_x_Mn_3‐x_O_4_ with immobilized Ir single atoms. The resulting Ir_1_/Ni_1.6_Mn_1.4_O_4_ has achieved overpotentials of 330 and 350 mV at 100 and 200 mA/cm^2^ of current densities in 0.5 m KOH with seawater. Enhanced activity can be attributed to the increased active sites, as well as the enhanced intrinsic activity of Ir single atoms. The catalyst has also shown great selectivity and anti‐corrosion properties. Such single‐atom systems are treated as a noble‐metal strategy because the dispersed noble atoms remain the principal active centers.

#### 4.3.2 Non‐Noble Metal‐Based Catalysts

While noble metals are the mainstay materials as water splitting catalysts, their overall scarcity and monofunctional behavior warrant the pursuit of earth‐abundant alternatives. Here, transition metals are commonly used in this area, with some derivatives reporting good bifunctional performance in both simulated and real seawater electrolytes. Table 4 highlights the performances of earth‐abundant electrocatalysts in various seawater electrolytes, to which the discussion is as follows.

**TABLE 4:** Summary of bifunctional electrocatalysts and their performances in various seawater electrolytes.

Material		Synthesis Method	Electrolyte	HER Overpotential at 100 mA/cm^2^ (mV)	OER Overpotential at 100 mA/cm^2^ (mV)	Cell Voltage at 100 mA/cm^2^ (V)	Stability	Refs.
MnCo/NiSe/NF	Potentiostatic + CV deposition		1 m KOH + Seawater	134.3	419.4 (500 mA/cm^2^)	1.86	200 h @ 500 mA/cm^2^	[143]
NiFeS/NF	Hydrothermal + ion exchange		1 m KOH + Seawater	217	226	1.67	100 mA/cm^2^ 50 h @100 mA/cm^2^) 25 h @500 mA/cm^2^	[146]
HCl‐treated NiFe foam	HCl solution corrosion		1 m KOH + 0.5 m NaCl	175	178	1.81 (500 mA/cm^2^)	300 h @ 100 mA/cm^2^	[155]
Se‐FeCo LDH/NF	Hydrothermal		1 m KOH + filtered seawater	220	295	1.92	24 h @ 100 mA/cm^2^	[151]
Cr_2_O_3_‐CoO_x_/Carbon fiber paper	Gas cation exchange + Thermodecomposition		Filtered seawater	N/A	N/A	1.89 (150 mA/cm^2^)	225 h @ 1.9 V	[22]
NiFeP nanowires (HER) + nanosheets (OER) /NiFe foam	Solvothermal		1 m KOH + seawater	130	147	1.62	500 h @ 100 mA/cm^2^	[148]
FeMoCoO/NF	Solvothermal + Thermal treatment		1 m KOH + seawater	250 (50 mA/cm^2^)	365 (50 mA/cm^2^)	1.59 (10 mA/cm^2^)	180 h @ 100 mA/cm^2^	[144]
S‐NiMoO_4_ nanorods@NiFe LDH/NF	Hydrothermal + Vulcanization + Electrodeposition		1 m KOH + seawater	220	315	1.73	20 h @ 100 mA/cm^2^	[156]
Ni@CNT‐Mo_x_C/Ni_2_P micropillars	Hydrothermal + Thermal annealing + Phosphidation		1 m KOH + seawater	65.9 (10 mA/cm^2^)	230 (10 mA/cm^2^)	1.56 (10 mA/cm^2^)	50 h @ 50 mA/cm^2^	[157]
MoN‐Co_2_N on N‐doped Carbon nanosheets	MOF template synthesis + annealing		1 m KOH + seawater	304	357	1.7	62 h @ 100 mA/cm^2^	[158]
NiCoP/NF	Hydrothermal + Phosphidation		1 m KOH + 0.1 m Xylose	152.6	130 (Xylose oxidation reaction)	1.57	N/A	[149]
Ni_2_P‐Fe_2_P/NF	In situ growth + ion exchange + phosphidation		1 m KOH + seawater	252	305	1.81	36 h @ 100 mA/cm^2^	[74]
Mo‐CoP_x_/NF	Hydrothermal + Phosphorylation		1 m KOH + seawater	82 (10 mA/cm^2^)	268 (10 mA/cm^2^)	1.61	100 h@ 10 mA/cm^2^	[159]
Fe_2_P/NiCoN/NiP/NF	Hydrothermal		1 m KOH + seawater	88	255	1.62	40 h @ 50 mA/cm^2^	[160]
CoNiFeMn Phosphoxide/NF	Electrodeposition		1 m KOH + seawater	289	432	1.54 (10 mA/cm^2^)	15 h @ 50 mA/cm^2^	[147]
Fe_3_O_4_/NiC_x_/Glassy carbon	Sol gel + annealing of NiFe PBA		1 m KOH + 0.5 m NaCl	480	308	1.66	30 h @ 500 mA/cm^2^	[145]
Ru/P‐NiMoO_4_ hollow nanorods/NF	Hydrothermal + Solution immersion + Phosphidation		1 m KOH + seawater + 0.5 m urea	N/A	230 (1000 mA/cm^2^)	1.73 (500 mA/cm^2^)	140 h at 100 mA/cm^2^	[150]
Ru_22_NiMoP_2_/NF	Hydrothermal		1 m KOH + filtered seawater	52 (10 mA/cm^2^)	242 (10 mA/cm^2^)	1.53 (10 mA/cm^2^)	20 h @ 10 mA/cm^2^	[161]
Pt_2_/Ni(OH)_2_)/NF	Hydrothermal		Alkaline seawater	76	N/A	1.45 (10 mA/cm^2^)	N/A	[152]
PtO_x_‐NiO_x_ nanosheets/NF	Heat treatment of Ni ZIF‐L + Dip coating (Pt)		1 m KOH + seawater	140	360	1.75 (50 mA/cm^2^)	9000 s at stepped current density	[153]
Pt‐CoFe LDH/NF	Electrodeposition + Dip coating (Pt)		1 m KOH + seawater	94	302	1.65	40 h @100 and 20 h @ 500 mA/cm^2^	[162]
FeNiCoMnRu@CNT high entropy alloy/Carbon paper	Chemical precipitation + annealing		1 m KOH + seawater	N/A	410	1.6	30 h @10 mA/cm^2^	[154]

##### 4.3.2.1 Metal–Nonmetal Alloys and Composites

**FIGURE 6:**
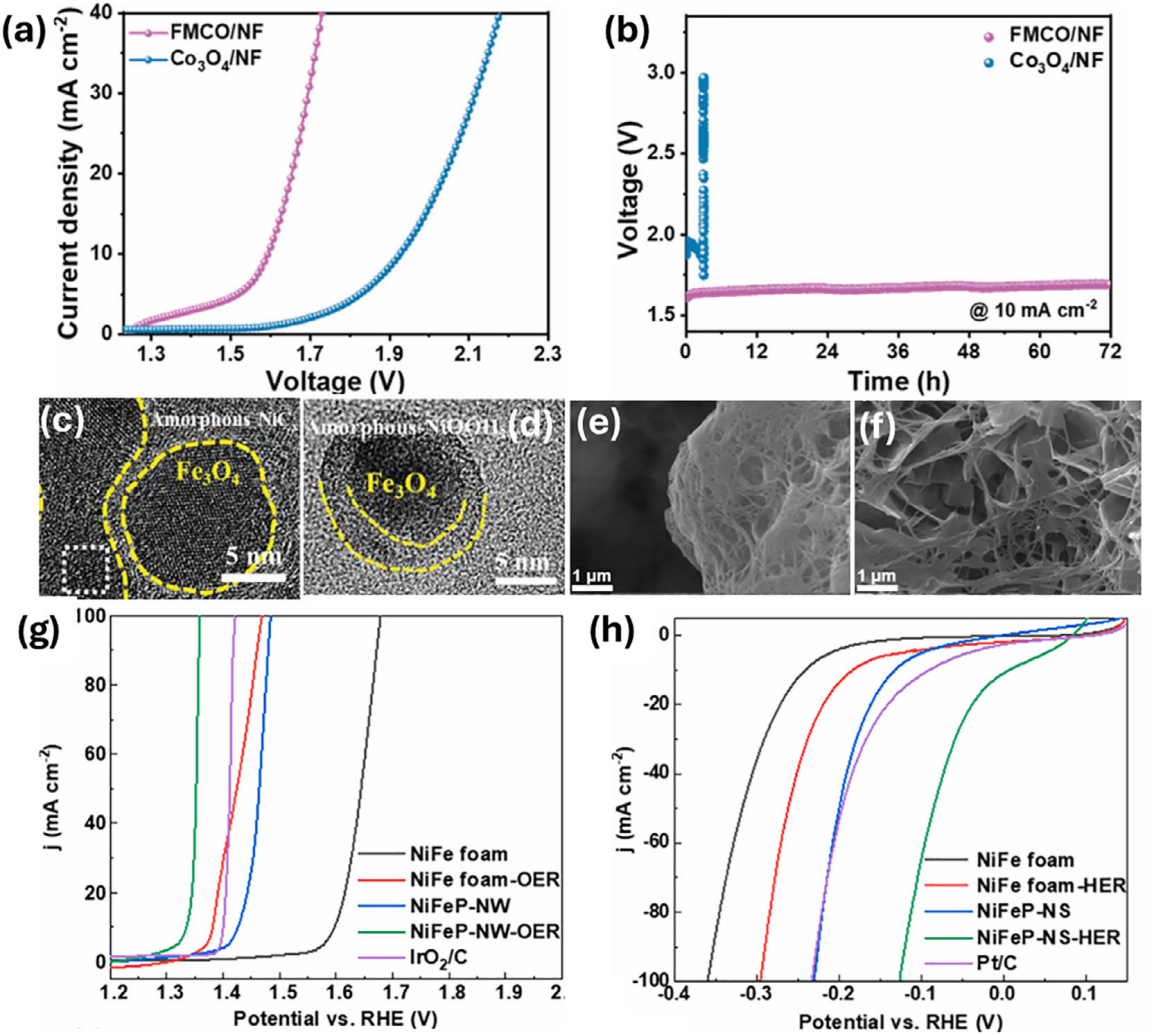
(a) LSV curves and (b) stability plot of FMCO/NF and Co_3_O_4_/NF catalysts in alkaline seawater. Reproduced with permission [144]. Copyright 2023, Elsevier. (c) TEM images of NiC_x_/Fe_3_O_4_ transformed to (d) NiOOH/Fe_3_O_4_ after alkaline seawater OER. Reproduced under terms of the CC‐BY license [145]. Copyright 2022, John Wiley and Sons. NiFeP catalysts of (e) NW and (f) NS morphologies synthesized using different solvothermal reaction times, (g) OER and (h) HER LSVs of the NiFeP catalysts in alkaline seawater (NP: nanoparticles, TF: thick flakes). Reproduced with permission [148]. Copyright 2022, Elsevier.

To achieve performance parity with noble metal catalysts, many studies agree on developing multimetallic variants of transition metal catalysts. This is usually carried out by either doping or alloying multiple metals during the synthesis process. Besides improving the catalyst's electrochemical surface area (ECSA), other motivations such as enhanced electron transfer and optimized HER/OER intermediate adsorptions are also highlighted as beneficial effects [143, 144]. In metal oxides like Co_3_O_4_, adding other transition metals like Fe and Mo is found to raise the binding energy of both Co^2+^ and Co^3+^ species, thus enhancing the OER intermediate adsorption for accelerated kinetics, which allows for lower cell voltage overpotentials in the FMCO/NF catalyst (Figure 6a). Meanwhile, Mo in the anodic reaction is transformed to MoO_4_2^−^ which can act as a chloride ion repellent, enhancing the FMCO/NF catalyst stability in alkaline seawater (Figure 6b) [144]. This property can also be manifested in core–shell structures, whereby the shell material serves as a protective layer to the core. Zhang et al. reported that meta‐stable NiC_x_ is in situ transformed into amorphous NiOOH during OER, which buffers the core Fe_3_O_4_ from the external chloride ions, while functioning as an OER active site (Figure 6c,d). The high presence of high‐valence Ni cations in this layer also changes the catalyst's OER mechanism from a conventional adsorbate evolution mechanism to a lattice oxygen oxidation mechanism, bypassing the need for adsorption of O_2_‐containing intermediates, leading to enhanced OER reaction kinetics. On the opposing electrode, NiC_x_ also improves the cathodic activity by transforming to Ni(OH)_2_ during HER, demonstrating the dual‐beneficiary effect of this layer for both reactions [145].

Looking beyond metal incorporation, the bifunctional performance of earth‐abundant catalysts can be enhanced by incorporating nonmetals into singular or multi‐metal materials. These elements can serve to lower the adsorption energy of HER or OER intermediates. For example, sulfur is also found to be a more preferential adsorption site for H‐atoms during HER, which reduces the adsorption free energy of NiFeS close to thermoneutral [146]. A more precise contributor to the lower adsorption energy effect can be traced to the formation of metal–nonmetal bonds. Zhang et al. explained that this is due to the large electronegativity differences between the elements. As a result, low electronegative elements donate electrons to elements with higher electronegativity, creating localized electric dipoles in which electron‐rich regions or elements better facilitate HER activity, with the inverse for OER [147]. It must be said that this effect is not confined to just metal–nonmetal combinations and can be applied to all metal derivatives too, though it is more pronounced on the latter. While some techniques such as electrodeposition allow for in situ incorporation of nonmetals during the initial synthesis, a majority require a secondary thermal‐induced synthesis using a nonmetal precursor. In the latter process, changing the reaction time and mass of the nonmetal precursor can affect the final catalyst morphology. Liu and co‐workers found that the morphology of NiFeP transitions from nanowires (NW) to nanosheets (NS) at increasing phosphorus mass loading and reaction temperature, affecting the overpotentials of both HER and OER (Figure 6e–h) [148]. Besides facilitating less energy‐intensive water‐splitting reactions, nonmetals also function as effective chloride ion repellents. Phosphorus and sulfur, when oxidized, respectively form electron‐rich phosphate and sulphate anions that can repel chloride ions [146, 148, 149, 150]. As such, metal phosphides and sulfides have generally been reported to have excellent stability in various seawater‐based electrolytes, highlighting their anti‐corrosive properties in this regard. Additionally, Se improves the overall water splitting performance of metal hydroxides like FeCo layered double hydroxides, while also enhancing the catalyst durability in alkaline seawater. As reported by Gong et al., the addition of Se anions (Se^2−^) can generate the protective aggregation of selenate around the catalyst [151].

#### 4.3.3 Design Strategies for Bifunctional Catalysts

##### 4.3.3.1 Lewis Acid and Electrolyte Modification

**FIGURE 7:**
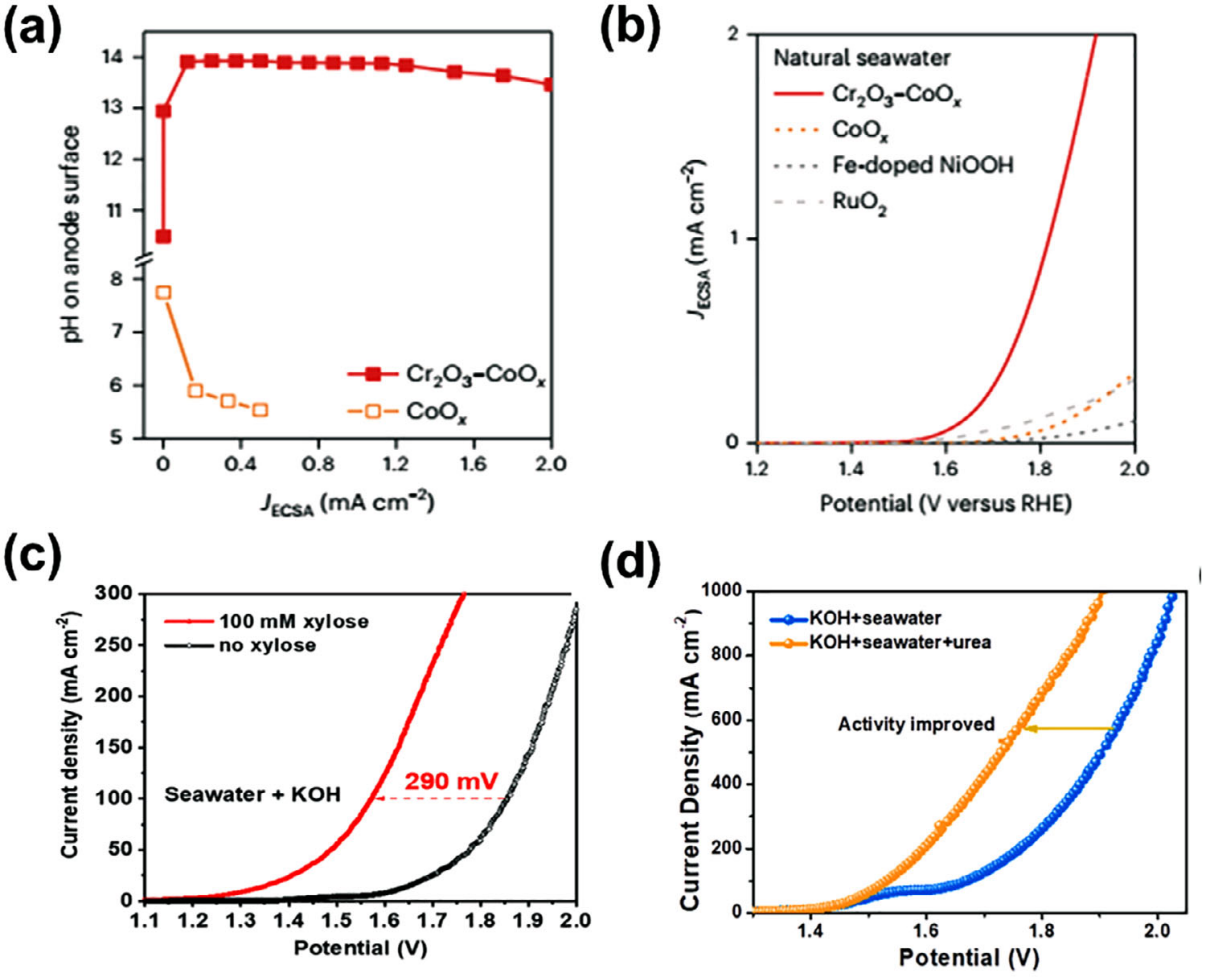
(a) Surface pH of Cr_2_O_3_‐CoO_x_ and CoO_x_ at different operating current densities and (b) their corresponding ECSA‐normalized OER LSVs in neutral seawater with RuO_2_ and Fe‐doped NiOOH. Reproduced with permission [22], Copyright 2023, Springer Nature Limited. (c) LSV of NiCoP/NF with and without addition of xylose. Reproduced with permission [149], Copyright 2023, The Royal Society of Chemistry. (d) LSV of Ru/P‐NiMoO_4_ hollow nanorods/NF with and without addition of urea. Reproduced with Permission [150], Copyright 2023, Elsevier.

Apart from incorporating non‐metals, another approach to prevent chlorine corrosion is to introduce Lewis acid layers onto an existing metal oxide catalyst surface. This layer serves to generate a localized alkalinity in said region (Figure 7a), which increases the surface concentration of OH^−^ until the saturation point, preventing the intrusion and interference of Cl^−^ ions in the OER reaction. A distinct advantage of this technique is the elimination of adding strong alkali into the seawater electrolyte, thereby introducing favorable cost‐saving benefits. The strong interaction between the Lewis acid and the in situ generated OH^−^ ions can also prevent the formation of unwanted particulates with other seawater cations (Mg^2+^ and Ca^2+^) at the cathode, which, together with the interfacial support between the two layers, also helps to improve the catalytic activity in OER (Figure 7b). To our knowledge, only Guo et al. have demonstrated such a catalyst design using Cr_2_O_3_ as a Lewis acid on CoO_x_, generating 1.87 V cell voltage at 1000 mA/cm^2^ in flowing natural seawater with 100 h stable activity at 500 mA/cm^2^ [22].

In some seawater electrolysis designs, the typical OER reaction at the anode is substituted with more thermodynamically favorable oxidative reactions. This is done to ensure the obtained overpotentials are substantially reduced to not cross the overpotential threshold (∼480 mV) that may initiate ClOR, preventing likely corrosion of the catalyst. This can be achieved by adding easily oxidizable materials into the seawater electrolyte. Xylose and urea have been reportedly added as replacement oxidizing agents. In both situations, earth‐abundant metal phosphides have been used as bifunctional catalysts, achieving lower overpotentials than what was otherwise achieved in OER reactions (Figure 7c,d) [149, 150]. While lower anodic overpotentials are generally achieved, an area of possible concern is the sluggish oxidative kinetics of some additives, notably urea with its six‐electron reaction versus the four‐electron OER. Overcoming this will require the design of even more efficient catalysts, earth‐abundant or otherwise.

##### 4.3.3.2 Trace Noble‐Metal Incorporation

While transition metals serve primarily as noble metal replacements in catalytic seawater splitting, it does not have to be an exclusive affair between both metal categories. Indeed, combining transition metals with trace amounts of noble metal can be beneficial for overall performance with minimal drawbacks from an economic perspective.

**FIGURE 8:**
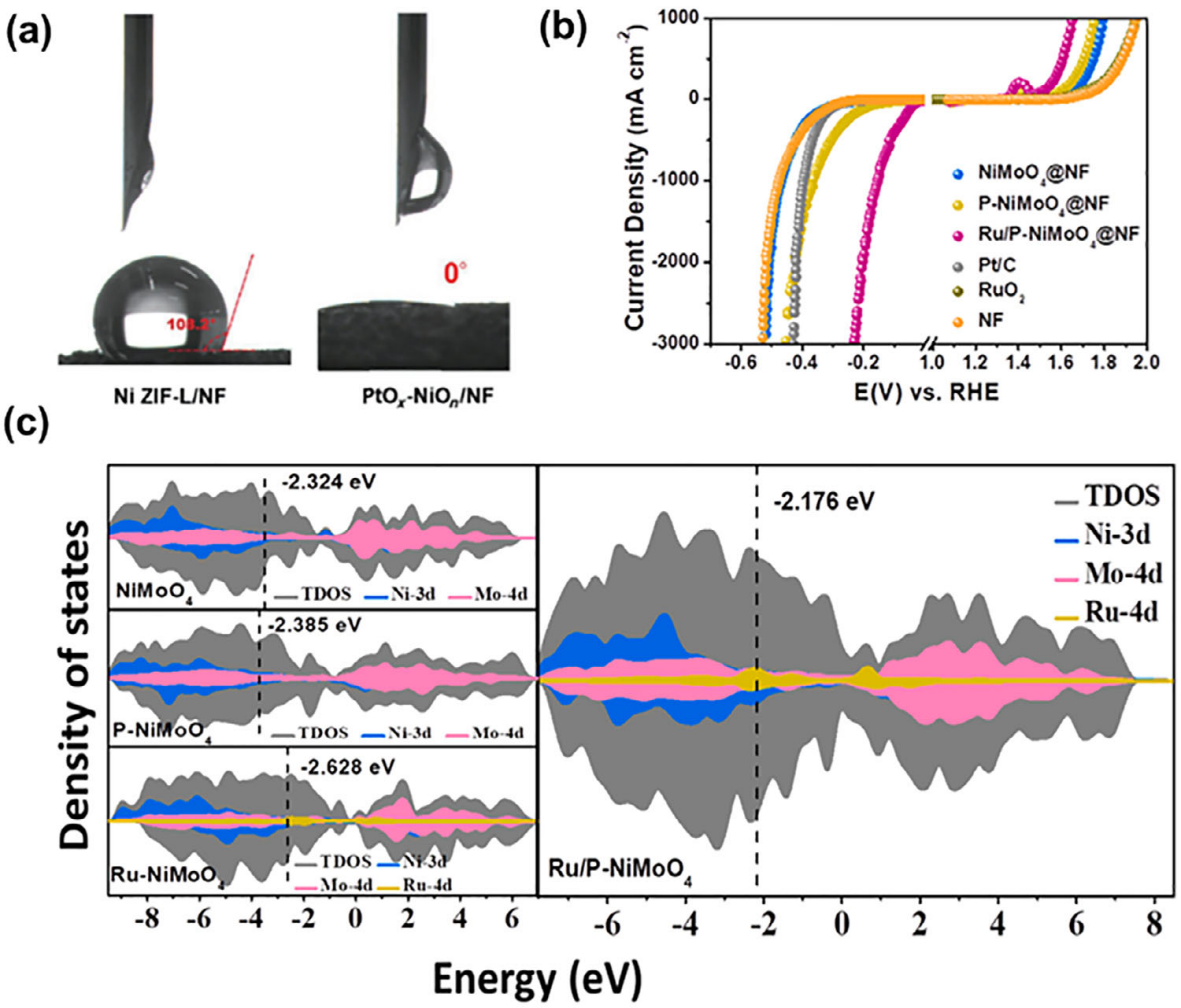
(a) Contact angles of Ni ZIF‐L/NF and PtO_x_‐NiO_n_/NF. Reproduced with permission [153], Copyright 2023, Springer Nature Limited. Comparison of Ru/P‐NiMoO_4_/NF with other catalysts, (b) LSVs in alkaline seawater and (c) Total and projected DOS. Reproduced with Permission [150], Copyright 2023, Elsevier.

In general, adding noble metals has a similar enhanced intermediate adsorption capability as when multiple transition metals are incorporated. Pt in particular helps to negatively shift the binding energy of Ni when bonded, which increases its affinity to adsorb H^*^ during HER activity [152]. At the same time, it also helps reduce the generation energy of O_2_ vacancies, thereby increasing their population on the catalyst surface. Crucially, Pt is super hydrophilic, which helps improve the adhesion of water molecules on the catalyst surface while facilitating rapid electron transfer across the catalyst–electrolyte interface (Figure 8a) [152, 153]. Ru also serves a similar role as an electron transport accelerator, while functioning as a more preferential OER active site [150, 154]. When incorporated into P‐NiMoO_4_ hollow nanorods, it is found by Guo et al. in Figure 8c that the density of states (DOS) at the Fermi level of Ru/P‐NiMoO_4_ becomes continuous (i.e., pseudo‐metallic), which shows the enhanced transport capability brought about by this incorporation. As a result, both HER and OER activities in seawater‐based electrolytes can be enhanced with this incorporation (Figure 8b) [150].

### 4.4 Major Challenges of Electrocatalysts

#### 4.4.1 Scalability Challenges

Scalability remains one of the most formidable barriers to the industrialization of DSE. While advancements have been made in developing high‐performance electrocatalysts, the transition from laboratory‐scale demonstrations to industrial‐scale applications presents multifaceted challenges.

Progress has been made to improve the scalability of seawater electrolysis catalysts. Yu's team presented a 5 cm × 5 cm Mo‐NiFeOOH/SSM OER electrode through surface etching of a stainless‐steel mesh. The introduction of MoO_4_2^−^ tunes the electronic structure and charge distribution of NiFeOOH, enhancing the binding of OER intermediates while inhibiting the competing absorption of Cl^−^. Together with the porous, hydrophilic surface that promotes electrolyte penetration and efficient bubble detachment, the resulting electrodes deliver strong corrosion resistance and excellent seawater OER performance. The catalyst achieved overpotentials of 274, 296, and 316 mV at current densities of 500, 1000, and 2000 mA/cm^2^ in a 6 m KOH seawater electrolyte. Moreover, the catalyst exhibited excellent durability, maintaining stable operation for more than 2000 h at 1000 mA/cm^2^ [163]. Fu et al. developed flexible Ni–P‐based nanoball electrocatalysts (NiP_x_@HA, HA is hydrophobic asbestos) that demonstrate excellent catalytic performance and durability for seawater electrolysis. Synthesized via electroless plating at 25°C on a flexible, anti‐corrosive hydrophobic asbestos substrate, the nanoballs feature strong chemical attachment, robust Ni_5_P_4_ crystalline modification, and rapid electron transfer. The catalyst achieved overpotentials of 208 mV for HER and 392 mV for OER at 200 mA/cm^2^ in alkaline seawater and sustained stability at 500 mA/cm^2^ for over 40 days. The versatile synthesis strategy supports multi‐elemental doping, flexible substrate use (e.g., filter paper, nickel foam (NF)), and scalability (22 cm × 22 cm), offering promising opportunities for industrial H_2_ production in corrosive electrolytes [164]. Instead of focusing on electrode size, Qiao's group directly investigated an industrial‐scale alkaline seawater electrolyzer using NiFe LDH as the anode. The system operated stably for over 100 h at 200 mA/cm^2^, reducing electricity consumption from 5.95 to 4.72 kWh/Nm^3^ and achieving a 20.6% energy saving [21]. Zhang and colleagues presented a bifunctional Fe_2_P/Ni_1.5_Co_1.5_N/Ni_2_P that required only 1.742 V to reach a current density of 500 mA/cm^2^ in 1 m KOH seawater electrolyte [160].

Despite these significant advancements, several challenges still hinder the large‐scale implementation of seawater electrolysis. The integration of scalable synthesis methods with industrial electrolyzer systems has yet to achieve the efficiency, stability, and cost‐effectiveness required for widespread adoption. These challenges include: (1) material and production costs: While nonprecious metal‐based catalysts such as NiFeP and NiP_x_@HA reduce reliance on costly noble metals, the cost of large‐scale synthesis, infrastructure, and operational requirements remains a barrier. For example, optimizing microstructures for enhanced performance at scale often requires additional processing steps, increasing production costs. (2) Long‐term stability in real seawater: many catalysts, including those highlighted above, have demonstrated impressive durability in simulated seawater environments. However, real seawater poses additional challenges, such as biofouling, corrosion from chloride ions, and contamination from impurities. These factors can significantly reduce the long‐term stability of catalysts under industrial operating conditions. (3) System integration: industrial seawater electrolyzers must integrate seamlessly with renewable energy sources for green H_2_ production. Variability in renewable energy supply, coupled with the need for robust and adaptable electrolyzer designs, adds complexity to system‐level deployment.

#### 4.4.2 Standardization and Benchmarking

Comparing the performance of different bifunctional catalysts for seawater splitting is complicated due to variations in test conditions. For instance, the choice of electrolyte can vary between simulated seawater and natural seawater. Natural seawater, collected from different oceans or coastal regions, exhibits variability in pH, conductivity, and composition, with many of these parameters remaining unanalyzed or unspecified. Furthermore, the treatment of natural seawater differs across studies, with some samples being filtered and others undergoing elemental removal. In contrast, simulated seawater typically consists of sodium chloride (NaCl), which fails to account for the impurities and complex compositions present in actual seawater.

Other critical parameters in electrochemical measurements include the current density used to evaluate overpotentials for catalytic activity. Studies often employ a wide range of values, such as 10, 100, 500, or even 1000 mA/cm^2^, making direct comparisons challenging. Similarly, the current density applied to assess stability also varies across studies, presenting comparable issues in standardizing performance benchmarks. The pretreatment of seawater can also make a difference to the performance of the electrolyzer [74].

From Table 5, it is evident that significant variability exists in the experimental conditions reported for catalysts in seawater splitting. This variability, including differences in electrolyte composition, current densities for activity and stability assessments, and seawater treatment methods, makes it difficult to establish a consistent baseline for comparison. These challenges highlight the need for standardized protocols to ensure consistent testing conditions and enable meaningful comparisons across studies.

**TABLE 5:** The different parameters used in studies on seawater catalysts.

Catalyst	Electrolyte composition	Seawater pretreatment	Current density of activity (mA/cm^2^)	Current density of stability (mA/cm^2^)	Refs.
Ni_2_P‐Fe_2_P	1 m KOH seawater. Natural seawater was collected from Galveston Bay, Galveston, Texas, USA (29.303°N, 94.772°W).	Natural seawater was stored in a bottle for approximately one week to allow visible sand and particles to settle, after which the clear supernatant was collected.	100, 500	100, 500	[74]
CoSe_2_‐NCF	1 m KOH + natural seawater. The seawater was extracted from China Bohai and filtered before being used (pH = 7.62, ionic conductivity *κ* = 72.5 mS/cm, *T* = 298 K).	Not mentioned	10	10, 100	[46]
(FeCoNi)OH‐S/NF	The alkaline seawater electrolyte was prepared directly using seawater (seawater taken from Dongjiang Bay, Tianjin) and KOH,	Removed the precipitation after standing.	10	10, 50, 100	[165]
Fe_2_P/Ni_1.5_Co_1.5_N/N_2_P	1 m KOH seawater	Not mentioned	100, 500	50	[160]
Fe_4_N/Co_3_N/MoO_2_	1 m KOH + seawater (seawater obtained from Zhanjiang in Guangdong gallery)	Not mentioned	1000	500, 1500	[166]
NiFeS/NF	1 m KOH + seawater; natural seawater was collected from Weihai, Shandong, China	Most of the magnesium and calcium salts were removed by first adding 3.4 g Na_2_CO_3_ to 500 mL of natural seawater before use	500	100, 500	[146]
NiFeP	The natural seawater from the Bohai Sea in China	No further purification.	100	100	[148]
Ni_3_N (Fe_2−2x_Co_2xP_/Ni_3_N)	1 m KOH seawater	Not mentioned	100, 500	100	[167]
FMCO/NF	1 m KOH + seawater, pH around 14; the seawater is taken from the Ningbo Zhoushan Port of China	Not mentioned	10	10	[144]
Fe‐NiS@HA	1 m KOH + 0.5 m NaCl		10	10	[168]

While a universally accepted protocol for DSE has yet to emerge, the mature freshwater electrolysis assessment protocol can be of reference, though not directly transferable. For freshwater electrolysis, McCrory et al. [169] reported a protocol for electrocatalytic assessment of the activity, stability, and FE. They suggested the reporting of overpotentials at a fixed geometric current density of 10 mA/cm^2^, together with electrochemical capacitance measurements, etc. This has become a widely used reference point. Voiry et al. [170] outlined important criteria for reporting the electrocatalytic performance of nanomaterials, recommending key parameters, such as specific surface area and Tafel slope. They also advised that during a three‐electrode electrocatalytic measurement, a well‐characterized commercial catalyst should be measured under the same electrochemical conditions as a reference for the researchers’ experimental setup for more concise comparison between different studies, for example, 20 or 40 wt.% Pt/C catalyst for HER.

Due to the greater complexity of DSE, the experimental configurations and reported results that require standardization demand more careful consideration. The choice of electrolyte is a representative example. Three types are most commonly utilized: natural seawater, alkalized seawater, and simulated seawater. Natural seawater is sampled directly from the ocean; however, the pretreatment applied to it needs to be specified, whether it is filtered or whether the precipitates are removed, etc. For alkalized seawater, a common alkaline concentration would need to be fixed. In practice, 1 m KOH is almost used widely enough to serve as a de facto standard, and formalizing it would be a natural starting point. For simulated seawater, the ionic composition would require a defined and complete recipe. Results reporting raises similar questions. Because DSE is oriented toward industrial application and coupling with renewable energy resources [171], the benchmark current density and operating temperature for evaluating activity might be raised toward industrially relevant conditions. Establishing a standardized DSE evaluation protocol will undoubtedly demand substantial effort, but the resulting benefit to the field would be invaluable.

## 5 Prospects of DSE Electrolyzers

While significant progress has been made in designing electrocatalysts for DSE at the laboratory level, the practical application at the industrial level still faces numerous challenges. Catalyst‐level advances translate into system performance only when the catalyst is matched to a compatible cell architecture. The integration of advanced electrocatalysts, ionic conductive membranes, and complex seawater electrolytes into the electrolyzer plays a critical role in the development of robust and efficient DSE systems. The selectivity and microenvironment control developed in Section 4 are realized depending on the membrane, electrolyte strategy, and operating regime in which the catalyst is placed, since these determine the local pH and ion environment at the interface as strongly as the catalyst's intrinsic properties do. Conventional electrolyzer configurations are usually incompatible with the complex and harsh seawater environment. The presence of numerous contaminants and salts in natural seawater leads to severe corrosion and scaling, which significantly degrade the lifespan and efficiency of the current electrolyzer. To bridge the gap between catalyst design and industrial application in the real world, this section systematically summarizes the current advancements and inherent operational challenges of conventional electrolyzer configurations in DSE. We further discuss the design of unconventional electrolyzers and highlight the design strategies in future DSE electrolyzers to mitigate the detrimental factors in the complex seawater environment.

### 5.1 Conventional Electrolyzer

**FIGURE 9:**
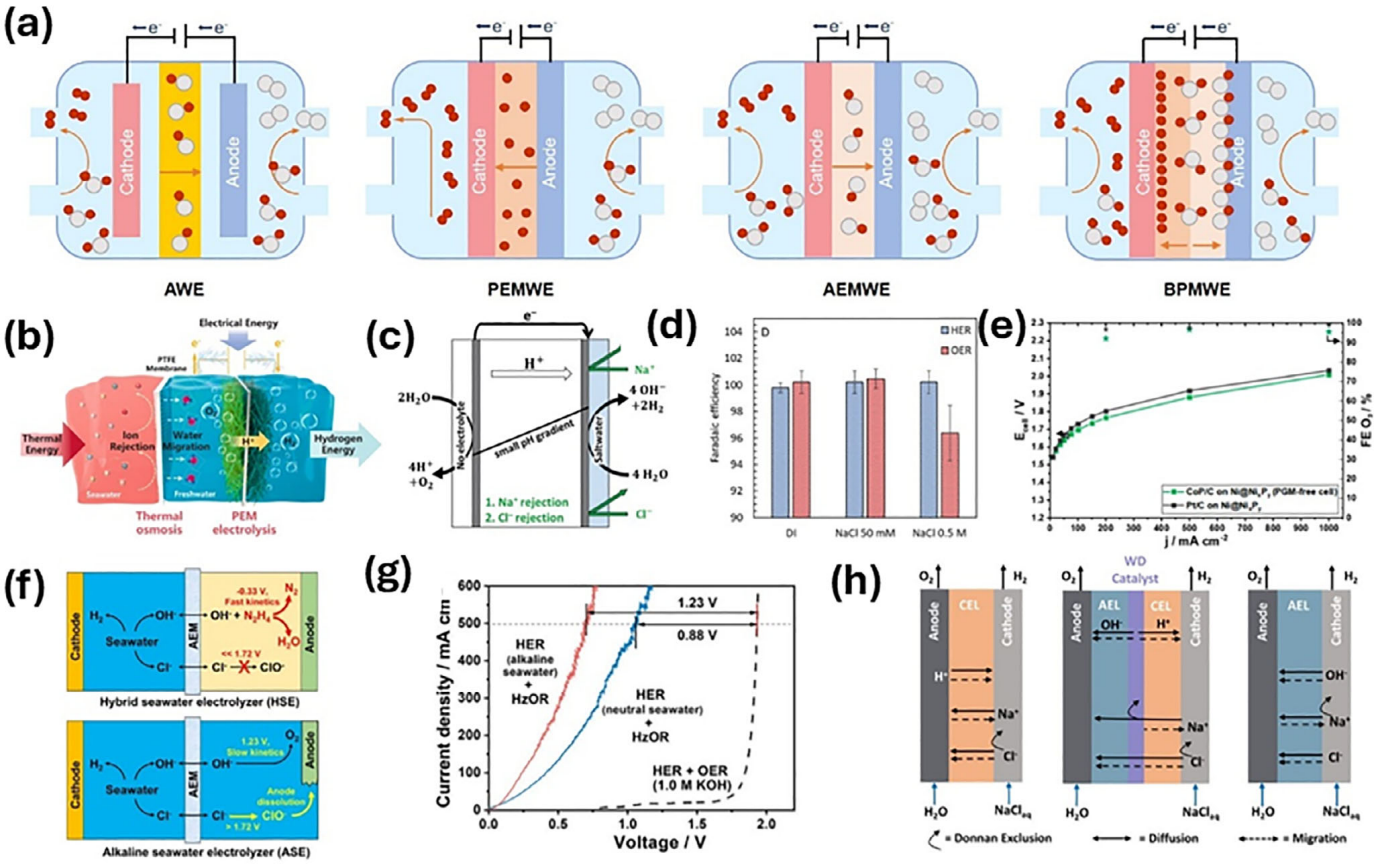
(a) Illustration of AWE, PEMWE, AEMWE, and BPMWE. (b) Schematic diagram of TO‐PEM integrated system. Reproduced with permission [180]. Copyright 2025, Elsevier. (c) Asymmetric feed PEM electrolyzer with vapor feed to the anode and saltwater for the cathode. (d) FE of asymmetric electrolyzer with different NaCl for the catholyte. Reproduced with permission [181]. Copyright 2021, Royal Society of Chemistry. (e) Polarization curves of all‐PGMs‐free AEMWE electrolyzer and Pt‐based benchmark MEA. Reproduced under terms of the CC‐BY‐NC‐ND 4.0 license [182]. Copyright 2023, American Chemical Society. (f) Schematic diagram of HSE and conventional seawater AEMWE. (g) Polarization curves of HSE with alkaline and neutral seawater compared with alkaline seawater electrolysis. Reproduced under terms of the CC‐BY license [183]. Copyright 2021, Springer Nature. (h) Schematic diagram of ion transport in PEMWE, BPMWE, and AEMWE. Reproduced with permission [66]. Copyright 2023, Elsevier.

Conventional electrolyzer configurations, including alkaline water electrolyzer (AWE), proton exchange membrane water electrolyzer (PEMWE), anion exchange membrane water electrolyzer (AEMWE), and bipolar membrane water electrolyzer (BPMWE), have been well established for pure water or alkaline water electrolysis (Figure 9a). However, the feasibility of these kinds of electrolyzer configurations under the seawater environment remains a challenge. Since conventional electrolyzer configurations require high‐purity electrolyte and stable operation conditions, the complex seawater environment will cause severe degradation, such as anode corrosion from competing ClOR, the formation of insoluble precipitate at the cathode side, and rapid membrane degradation. A thorough understanding of current bottlenecks in conventional electrolyzers in DSE is vital, as they provide fundamental motivation for future innovative DSE electrolyzer system designs.

#### 5.1.1 Alkaline Water Electrolyzer

As the most mature and earliest developed electrolyzer technique [172, 173], AWE has been extensively investigated for seawater electrolysis. The simple and robust cell construction of AWE, which consists of an anode and a cathode separated by a diaphragm, guarantees high operational reliability, low maintenance costs, and an impressive lifespan up to 10 years [174]. Furthermore, the ability to use inexpensive, earth‐abundant Ni‐based electrodes significantly lowers the cost of the electrolyzer [175]. Numerous studies have been dedicated to the design of laboratory‐level catalysts for alkaline seawater electrolysis.

Typically, industrial AEWs operate under high temperatures (70°C–90°C) and high concentrations of alkaline solutions (20–30 wt.% KOH) [176, 177], which are far more rigorous than the ambient temperature and 1 m KOH conditions in laboratory‐scale research. When applied to alkaline seawater electrolysis, the high pH environment not only enhances the sluggish kinetics of the OER but also significantly improves its selectivity by suppressing the competing ClOR at the anode. However, such a high alkalinity environment poses a critical challenge for the cathode. However, the high concentration of OH^−^ in alkaline solution can easily combine with metal cations in seawater (primarily Ca^2+^ and Mg^2+^) to form insoluble hydroxide precipitates (Mg(OH)_2_ and Ca(OH)_2_) [178], causing severe cathode scaling and diaphragm clogging, leading to performance degradation and long‐term instability of AWE in DSE. The high‐alkalinity electrolyte can also react with CO_2_ from the air, forming insoluble carbonate precipitates (e.g., K_2_CO_3_), which can further compromise the structural integrity and flow dynamics of the electrolyzer. Beyond these seawater‐specific challenges, AWEs also suffer from inherent operational bottlenecks that limit their large‐scale DSE application. The slow start‐up/shutdown responses [174], especially when coupled with intermittent renewable energy sources, significantly hinder their practical applications. Also, the relatively low operating current density and large device components compared with a compact membrane electrode assembly (MEA) system make it less competitive in DSE.

As discussed in the previous section, the majority of research in alkaline DSE primarily focuses on modifying catalysts and electrodes. However, in addition to catalyst development, optimization of the electrolyzer system plays a crucial role in enabling stable and practical alkaline DSE operation. An effective strategy in this regard is integrating electrolyte pre‐treatment and management systems with AWE. By integrating simple setups such as filtration and electrolyte circulation, the stability issues caused by the formation and accumulation of insoluble precipitates can be significantly mitigated. For instance, Gendel et al. [179] designed a new system combining pre‐separation of Ca^2+^ and Mg^2+^ in natural seawater by nanofiltration, and following mixing with the high‐concentration NaOH solution, enabling chlorine‐free seawater electrolysis. The concentrated OH^−^ can effectively suppress the ClOR, while the accumulated NaCl precipitates can be transported to the outside through the circulatory system. This system demonstrated 12 days of stable operation without Cl_2_ or chlorate production under a current density of 467 mA/cm^2^. Although this system‐level solution can effectively mitigate cathode precipitation and undesired ClOR, it suffers from the disadvantages of a large setup and increased overall cost.

#### 5.1.2 Proton Exchange Membrane Water Electrolyzer

Compared to AWE, as the representative of the MEA configuration, PEMWE offers a significantly more compact electrolyzer structure, which substantially lowers the interfacial resistance and operational cost. Thus, enabling PEMWE to operate under high current density with rapid start‐up/shutdown capabilities and high‐purity products, and suitable for coupling with intermittent renewable energy sources [184, 185]. These features make PEMWE one of the most promising techniques for industrial‐scale water electrolysis. However, the stable operation of PEMWE is basically reliant on the high‐purity water feed, which is supplied to the anode and decomposes into O_2_ and protons. The proton migrates through the Nafion membrane to the cathode and undergoes HER. This inherent dependency on pure water raises formidable challenges for PEMWE when applied to natural seawater electrolysis. Although the acidic environment at the cathode can effectively suppress the formation of insoluble hydroxide, the ClOR can severely affect the anodic reaction under acidic conditions, leading to the degradation of the catalysts’ stability and selectivity, which brings great challenges for catalyst design. The low pH environment and high chloride concentration electrolyte also cause aggressive corrosion, leading to irreversible damage to catalysts and cell components. Furthermore, the vulnerable Nafion membrane could be damaged by impurities and contaminants in seawater, and the metal cations (e.g., Ca^2+^, Mg^2+^, Na^+^, and K^+^) in natural seawater possess a high affinity for the sulfonic acid groups in the membrane and would compete with protons [186], block the H^+^ transport channels, result in the increase of ohmic losses, and decreased membrane lifespan.

These factors make the stable and long‐term operation of PEMWE in DSE practically unfeasible; while a significant breakthrough in catalyst design is still urgent, the optimization of the PEMWE system and innovative electrolyte management may be more promising. Recently, an integrated thermal osmotic‐PEM (TO‐PEM) setup, consisting of a PEM electrolyzer and a thermal osmosis module with a hydrophobic polytetrafluoroethylene (PTFE) membrane as shown in Figure 9b, was proposed by Huang et al. [180], combining the advantages of indirect seawater electrolysis (ISE) and DSE. The setup utilizes low‐grade thermal energy to create a temperature gradient across the PTFE membrane, enabling phase‐change mass transfer of natural seawater to in situ supply freshwater to the electrolyzer. This compact electrolyzer system prevents direct contact between the core components of PEMWE and impurities from seawater, while maintaining a stable pure water supply. The system demonstrates exceptional durability for 108 h with a H_2_ production rate of 217.7 mL/h with a natural seawater supply from the Bohai Sea. Despite realizing direct PEM seawater electrolysis within a single setup, the system's high cell voltage and its reliance on an external thermal source present significant drawbacks in terms of energy efficiency and cost. Another approach using asymmetric electrolyte feeding for PEMWE (Figure 9c) was proposed by Logan et al. to minimize the competing ClER at the anode [181]. The salt electrolyte was used as the cathodic electrolyte while a humidified gas stream was supplied to the anode. Such an asymmetric electrolyte supply system can effectively suppress the ClER in PEMWE and prevent the sodium diffusion across the PEM. The selectivity of the PEM can effectively reject Cl^−^ to the anode side, enabling a FE of 100% in 50 mm NaCl and slightly decreased to 96% in 0.5 m NaCl (Figure 9d). This setup obtains stable operation using synthetic seawater up to 40 h at 60 mA/cm^2^ with common electrocatalysts for PEMWE.

#### 5.1.3 Anion Exchange Membrane Electrolyzer

As one of the most promising water electrolysis techniques, AEMWE synergistically combines the advantages of both AWE and PEMWE. Similar to PEMWE, the MEA‐based compact structure of AEMWE minimizes the gas crossover and enables high current density operation at the same time [187, 188]. The conduction of OH^−^ in AEM and a low‐alkalinity operation environment allows AEMWE to use non‐precious metal catalysts, which makes it more compatible with natural seawater [189]. Also, its alkaline operation conditions can inherently suppress ClOR and favor anode selectivity for OER. However, the transition of AEMWE from laboratory techniques to large‐scale DSE is impeded by some practical challenges. The inherent mechanism of AEM enables it to conduct Cl^−^ and OH^−^ at the same time [25]. When operating under alkaline seawater feed, the competitive Cl^−^ transport will lead to the degradation of membrane selectivity, leading to an increase in interfacial resistance. Moreover, the crossover of Cl^−^ to the anodic side can trigger the competitive ClER and lead to the corrosion of anode materials. Another significant problem is that the high pH operation condition of AEMWE makes the system susceptible to severe scaling, as the insoluble precipitation of Ca^2+^ and Mg^2+^ in the cathodic chamber not only degrades the cathode catalysts but also blocks the AEM, ultimately leading to the collapse of the electrolyzer.

Despite these formidable challenges, AEMWE is still the most promising electrolyzer configuration for DSE. The ability of AEMWE to eliminate the reliance on noble metal‐based catalysts makes it extremely attractive to both industry and researchers. For example, Strasser et al., [182] demonstrated an asymmetric‐feed electrolyzer for DSE using platinum‐group metals (PGMs) free materials for all components of AEMWE, and catalysts with CoP/C‐based catalysts were used as the cathode and NiFe‐LDH as the anode. To pursue an all‐PGMs‐free electrolyzer system, a Ni‐based porous transport layer (PTL) was used for both the anode and cathode sides to improve the performance. They find that the asymmetric feed operation (alkaline seawater for anode and dry N_2_ for cathode) can effectively improve the corrosion resistance of the electrolyzer compared with a conventional Pt‐based MEA, and the dry cathode also enables high‐purity H_2_ production. As a result, this setup achieves a high current density of 1000 mA/cm^2^ below 2 V_cell_ at 60°C (Figure 9e), demonstrating the great potential of AEMWE in DSE. However, although the dry cathode can prevent the formation of precipitation, the anodic side still suffers from the competing ClOR and the degradation of the membrane due to the existence of Cl^−^. Another alternative approach is replacing the kinetically sluggish OER with another reaction to lower the cell voltage and avoid harmful ClOR. Qiu et al. [183]. demonstrate a HSE splitting coupling hydrazine degradation in conventional AEMWE (Figure 9f), using alkaline seawater as cathodic electrolyte. The generated OH^−^ in HER transports to the anode by AEM and supplies for the hydrazine degradation. When the cathodic chamber was fed with natural seawater, it showed a current density of 500 mA/cm^2^ at a low cell voltage of 1.05 V (Figure 9g), which is 45.6% lower than conventional alkaline seawater electrolysis, and achieved 85 h of stable operation at 300 mA/cm^2^. The cell voltage of this hybrid electrolysis system can be further reduced to 0.7 V at 500 mA/cm^2^ when using 1 m KOH seawater.

Because replacing the OER with an alternative anodic reaction modifies the reaction scheme rather than the cell architecture, this strategy is more accurately classified as hybrid seawater electrolysis and will be discussed in detail in Section 5.2.4.

#### 5.1.4 Bipolar Membrane Electrolyzer in DSE

While BPMWE is an emerging concept in water splitting, bipolar membranes themselves are well‐established in other fields like CO_2_ reduction [190, 191] and electrodialysis [192, 193]. This established technology provides a distinct strategic pathway for overcoming the inherent challenges of DSE. A BPMWE system contains a cation exchange layer (CEL) and an anion exchange layer (AEL) [41, 66], where the water molecules dissociate into protons and hydroxide at the junction of membranes and further transport to the cathodic and anodic chambers. Such a configuration can create an asymmetric pH environment in a single electrolyzer, which provides low pH conditions at the cathode side to favor the HER and suppress the undesired metal cation precipitation, and high pH conditions at the anode side to increase the selectivity for OER and minimize the competing ClOR. Also, the directional ion transport in BPM can effectively suppress the transport of other impurity ions in seawater (e.g., Cl^−^ crossover) (Figure 9g), enhancing the stability and durability of the system. The successful application of BPMMWE in DSE demonstrated stable operation at 250 mA/cm^2^ for more than 100 h with natural sea feed to the cathode side, and the FE of free chlorine formed during operation is only 0.005% [66]. A more advanced BPM configuration has recently been demonstrated by integrating a porous solid electrolyte (PSE) chamber between the CEL and AEL, enabling simultaneous seawater electrolysis and in situ electrodialysis desalination. In this multi‐chamber design, acidic HER and alkaline OER are spatially decoupled while salt ions are continuously removed, achieving nearly 100% coupling efficiency and stable operation for 360 h in real seawater [194].

Although BPMWE shows potential to solve the current challenges in DSE, it requires an additional power supply to ionize the water in the membrane, and the cell voltage is much more sensitive to impurities in the electrolyte, which significantly increases the energy consumption. The complex membrane structure undoubtedly increases its vulnerability to impurities in seawater, leading to the fast degradation of the membrane. As an emerging technology for DSE, significant research is still required to address the long‐term stability and degradation mechanism of BPM, and further optimization of the performance is urgently needed.

Overall, although numerous strategies have been applied to conventional electrolyzers in DSE, such as integrating a pretreatment system, using the asymmetric feed model, and structural optimization, the performance of these electrolyzers in DSE is still not comparable with the operation in pure water or alkaline solution. As these conventional electrolyzers are primarily designed to work under certain electrolytes, the incompatibility with the seawater environment causes the degradation and final failure of the electrolyzer. The design of high‐durability and stability catalysts is crucial for DSE, while system‐level strategies are also important, especially when the bottleneck of catalyst design arises. The large amount of organic and inorganic impurities in seawater also brings a great challenge to membrane stability; thus, strategies to protect the membrane and further investigation of the membrane degradation mechanism are urgently needed.

### 5.2 Unconventional Electrolyzer

As conventional electrolyzer configurations were not designed for DSE, a promising pathway is to develop novel electrolyzer configurations and membranes specific to complex seawater conditions. In this section, we will explore several unconventional electrolyzer configurations for the next‐generation DSE, with emphasis on understanding the fundamental design concept in these systems.

#### 5.2.1 Electrolyzer Integration With Seawater Pretreatment Techniques

**FIGURE 10:**
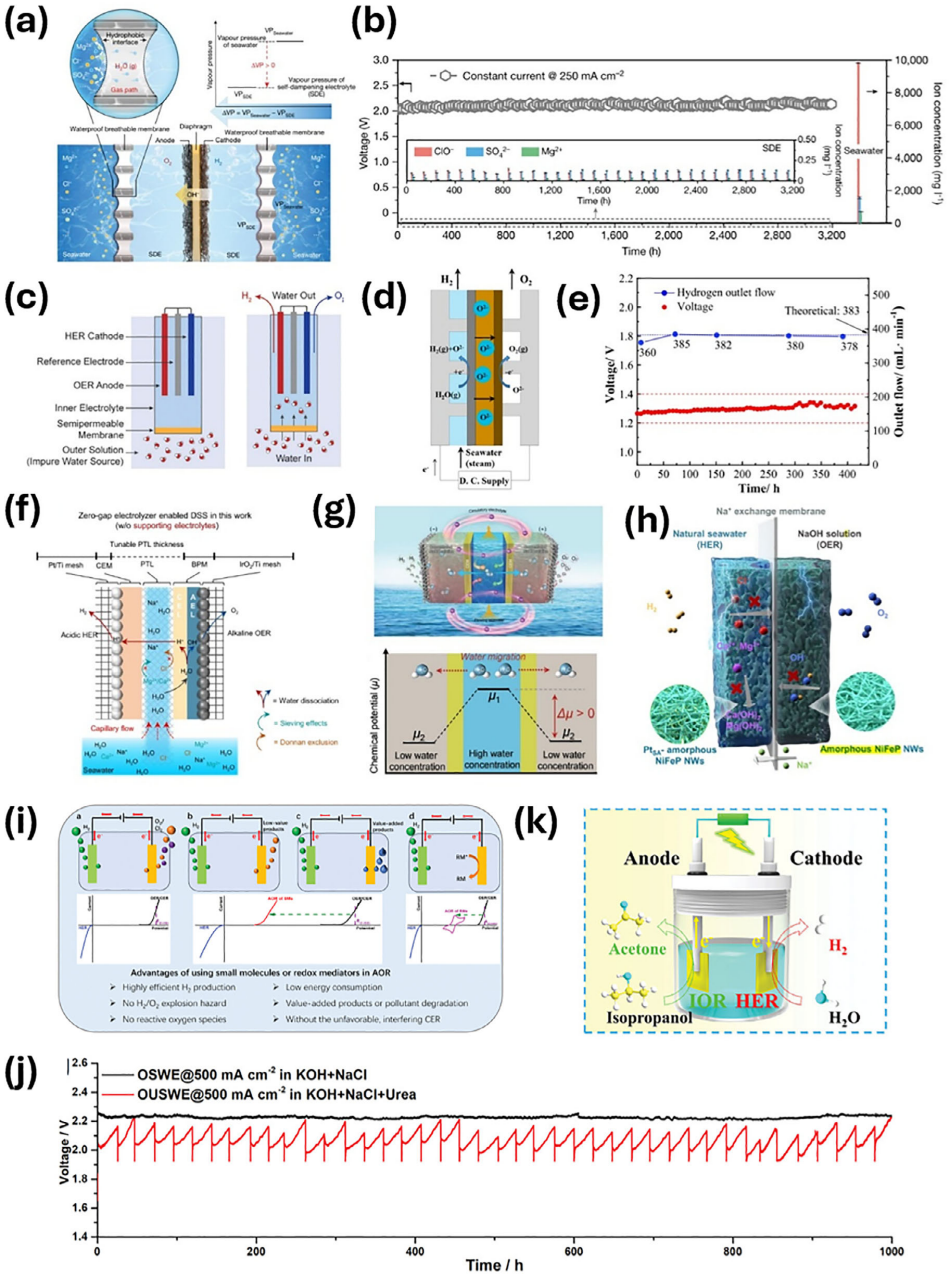
(a) Schematic diagram of the phase‐transition mechanism for water purification and the electrolyzer configuration. (b) Long‐term operation of scaled‐up SES at 250 mA/cm^2^ and the impurity ion concentration during the operation. Reproduced with permission [195]. Copyright 2022, Springer Nature. (c) Illustration of FOWS setup configuration and outflux and influx during operation [197]. (d) Structure of SOEC. (e) Long‐term operation of SOEC at 200 mA/cm^2^ and H_2_ production rate. Reproduced with permission [200]. Copyright 2021, Elsevier. (f) Schematic diagram of a zero‐gap electrolyzer consisting of a CEM and a BPM. Reproduced with permission [84]. Copyright 2024, John Wiley and Sons. (g) Configuration of a three‐compartment electrolyzer with a dual‐CEM and the illustration of water migration driven by a concentration gradient. Reproduced under terms of the CC‐BY license [85]. Copyright 2024, John Wiley and Sons. (h) Schematic diagram of a pH asymmetric electrolyzer with Na^+^ exchange membrane. Reproduced under terms of the CC‐BY license [61]. Copyright 2023, Springer Nature. (i) Schematic diagram of four different water electrolysis: (a) coupling HER with OER, (b) coupling HER with the AOR of SMs that generate low‐value products, (c) HER paired with the AOR of SMs that generate high‐value products, (d) HER paired with the AOR of redox mediators. Reproduced with permission [203]. Copyright 2023, John Wiley and Sons. (j) Chronopotentiometric curves of the NiFeS/NiFeP electrode pair at the current density of 500 mA/cm^2^. Reproduced with permission [204]. Copyright 2022, John Wiley and Sons. (k) Schematic illustration for isopropanol‐assisted water splitting system. Reproduced with permission [205]. Copyright 2023, John Wiley and Sons.

Compared with ISE, which requires additional facilities and energy input to purify seawater, integrating in situ seawater purification techniques into the electrolyzer seems more effective and economically affordable. As mentioned in the previous section, using components such as a selective membrane and an advanced electrolyte management system, this strategy has demonstrated promising results. Under such a design concept, a breakthrough seawater electrolysis system (SES) design was proposed by Shao et al. [195] for the first time in 2022, and successfully demonstrated industrial‐level operation using natural seawater at 250 mA/cm^2^ for 3200 h (Figure 10b). The system utilizes a hydrophobic porous PTFE membrane as the waterproof breathable membrane (WBM) to isolate the outer side natural seawater and concentrated KOH within the electrolyzer (Figure 10a). This PTFE‐based membrane impedes the penetration of seawater and contaminants, while the different water vapor pressures between the two sides of the membrane force the seawater to evaporate and diffuse through the gas path inside the membrane to the electrolyzer. This self‐driven phase transition migration of seawater can continuously supply purified water for electrolysis without requiring an additional power supply for treatment and effectively blocks all impurity ions. More importantly, such a configuration provides an economically affordable approach for large‐scale DSE; the energy consumption during long‐term operation is only 5 kWh/Nm^3^ H_2,_ which is comparable with the traditional alkaline electrolysis using pure water, and the traditional catalyst materials without further modification can be directly used in this system.

Another representative technique is forward osmosis water‐splitting (FOWS) setup. Unlike reverse osmosis, which is widely used in seawater treatment [6, 19, 196], the FOWS technique doesn't need an extra energy supply for the purification process. Similar to the aforementioned WBM system, the FOWS can also realize the in situ water extraction from saline water. This approach was first proposed by Nocera et al. [197] in 2021; as shown in Figure 10c, the setup consists of a conventional single electrochemical electrolyzer and is separated from impure water by a semipermeable membrane. The inner electrolyte is 0.8 m NaP_i_ buffer solution, while the electrolyzer is surrounded by 0.6 m NaCl solution. Leveraging the concentration gradient (0.2 m) between impure water and concentrated electrolyte through passive forward osmosis, this setup can continuously supply pure water to the electrolyzer. The water provided by forward osmosis will be consumed by electrolysis; thus, the concentration gradient can be maintained. This system achieved 48 h of stable operation at 250 mA/cm^2^, and was further extended to natural seawater in 2022 [198]. These integrated online purification systems provide a promising solution to DSE as they can directly circumvent rigorous ClOR and scaling issues, while the practical application still faces some limitations. As the purification process highly relies on the membrane, the reliability is critically dependent on the stability and long‐term selectivity of the membrane to isolate contaminants and multiple ions in seawater; any degradation or failure will directly impact the system's lifespan. Another challenge is that the slow passive purification rates may not be able to match the water consumption in a practical industrial electrolyzer, which will limit the overall energy efficiency, as these setups usually utilize spontaneous purification processes like osmosis or vapor pressure gradients. Moreover, the risk of leaking the concentrated internal electrolytes poses a significant environmental and safety concern.

#### 5.2.2 Solid Oxide Electrolysis Cell

Compared with the aforementioned low‐temperature (<100°C) water electrolyzer, SOEC operates at high temperature, utilizing proton‐conducting solid oxide (450°C–600°C) or oxide‐ion‐conducting solid oxide (700–850°C) and displays unique advantages for DSE. Integrating with thermal energy sources (e.g., nuclear or solar), SOEC possesses higher electrical efficiency and better reaction kinetics. When applied in DSE, SOEC can utilize the gaseous water evaporated from natural seawater; the water molecules are transported into the cathode for HER, and the resultant O^2−^crosses the membrane to the anode for OER. Such a configuration avoids any impurities from seawater, circumventing the effect of undesired ions (Cl^−^, Ca^2+^, Mg^2+^) in conventional electrolyzers, leading to an extended system lifespan and durability. The largest advantage of SOEC in DSE is that the operation with seawater gives almost the same performance as pure water, which was demonstrated by Chan et al. [199] in 2017, showing the great potential of SOEC in DSE. The degradation caused by seawater contamination is almost eliminated in SOEC. However, the practical application of SOEC in DSE remains scarce. The harsh high‐temperature conditions demand high‐temperature durable materials and bring extra challenges to electrolyzer design. Singhal et al. [200] demonstrate a SOEC setup based on double‐sided cathodes with natural seawater steam feed (Figure 10d) to verify the feasibility in DSE. The setup successfully operates at 200 mA/cm^2^ for 420 h with 183 mL/min H_2_ production at 750°C. The overall potential slightly increased from 1.265 to 1.316 V after 420 h of operation (Figure 10e) with an energy efficiency of 72.47%. Nevertheless, this study also exposes some practical issues during the operation. The intake pipe was completely blocked by sea salt after the operation. While the electrolyzer performance remains stable, it still demonstrates that a trace amount of salt can be carried by the steam from seawater, posing a long‐term degradation risk for the whole system. Periodic maintenance and novel electrolyzer structural design are necessary for future large‐scale applications.

#### 5.2.3 Novel Electrolyzers for Ion Management

The complex seawater condition poses distinct challenges to both the anode and cathode. As we discussed before, most of the current schemes for DSE are still based on the modification of conventional electrolyzers. Also, most of the current DSE techniques still need a supporting electrolyte to help extract water from seawater and accelerate the following water splitting, which usually leads to an extra energy consumption of up to 12.5% when using a typical KOH electrolyte (US$1000 t^−1^ KOH) [201]. To address this issue, Cui et al. [202] proposed a zero‐gap electrolyzer configuration integrating a monovalent‐selective cation exchange membrane (CEM) and a bipolar membrane, which can operate with natural seawater feed without the use of a supporting electrolyte. As shown in Figure 10f, the BPM is directly adhered to the anode, where seawater can be transported into the electrolyzer by capillary force, and the CEM can reject the diffusion of Cl^−^. At the same time, the cathode‐side CEM can protect the cathode from Mg^2+^ and Ca^2+^ while allowing H^+^ transport. This CEM/BPM system demonstrates stable operation for 120 h at 100 mA/cm^2^ with only natural seawater feed, successfully regulating undesired ions (Ca^2+^, Mg^2+^, Cl^−^) and the water molecule pathway to reach 100% FE. This configuration paves a new design concept to cope with the impurity ions in DSE. However, the excessively high cell voltage brings economic considerations, and the complex electrolyzer configuration causes difficulties in setup and maintenance. Furthermore, the long‐term stability of membranes remains problematic as the selectivity of CEM could decrease during operation and lead to the failure of the system.

A three‐compartment electrolyzer configuration coupled with a dual‐CEM was also proposed by Cui et al. [85] and co‐workers in 2024. The monovalent CEMs separated two electrolyte chambers concentrated with NaOH (Figure 10g), allowing a continuous water supply without disturbance of Ca^2+^/Mg^2+^ and Cl^−^, and the ionic neutrality can be maintained by selective permeation of monovalent cations. By circulating the NaOH electrolyte, the pH and concentration difference can be eliminated during the operation to ensure a sufficient concentration gradient for water transport. The relatively stable environment enables the system to utilize Ni foam as the catalyst, delivering 1 A current over 100 h. CEM has shown great potential in these novel electrolyzers for DSE, although the operating voltage of these systems is usually higher than conventional configurations. Li et al. [61] designed a pH‐asymmetric electrolyzer with an Na^+^ exchange membrane (Figure 10h) to significantly lower the cell voltage compared with other CEM‐based electrolyzers. Natural seawater was directly supplied to the cathode side, while the anodic chamber was provided with NaOH solution. Na^+^ exchange membrane can block the transport of Cl^−^ to the anode, preventing the harmful ClOR, and the near‐neutral seawater environment at the cathode can suppress the precipitation of metal cations. The system demonstrates an ultra‐low potential of 1.46 V at 100 mA/cm^2^ and 1.66 V for 400 mA/cm^2^ at 80°C. This high performance can be ascribed to the chemical potential difference between cathodic and anodic electrolytes with different pH conditions. But still, as we mentioned before, the stability of CEM in practical operation remains problematic, which could cause system failure when the membrane degrades. The conduction of Na^+^ in the circulatory system may also lead to the accumulation of NaOH, which can pose challenges to electrolyte management.

#### 5.2.4 Hybrid Seawater Electrolyzer

A fundamentally different route to avoid the chlorine problem is to abandon the OER altogether. In hybrid seawater electrolyzers (HSEs), the sluggish four‐electron OER at the anode is replaced by the anodic oxidation reaction (AOR) of a small molecule (SM) or a redox mediator. The cell voltage and electrical energy demand are reduced drastically. The ClER is suppressed because the AOR proceeds well below the chlorine onset. The operational safety is also improved because there is no more H_2_/O_2_ mixture with explosion hazard [203].

The SMs for AOR can be categorized into two types. The first comprises sacrificial agents regarded as environmental pollutants, such as urea and hydrazine. In this case, HSE produces H_2_ via the HER and simultaneously enables waste treatment through the AOR [203]. Liu et al. [204] reported a self‐supported NiFeSP nanotube array electrode as a bifunctional catalyst for the anodic UOR and the cathodic HER. The abundant NiFeS/NiFeP heterointerfaces and defective sites enhanced the catalytic activity, enabling an overall urea‐mediated seawater electrolyzer that required only ∼2 V to reach 500 mA/cm^2^, and operated stably for 1000 h with negligible degradation. The second comprises feedstocks that can be converted into products of high economic value, such as alcohol and biomass‐derived SMs. In this case, HSE yields two valuable products [203]. Wang's group [205] developed an atomically thin defect‐rich PdIr bimetallene nanoribbons (PdIr BNRs) catalyst for an isopropanol‐assisted seawater electrolysis system. Comprehensive physicochemical characterization revealed that PdIr BNRs present a high density of atomic sites alongside abundant structural defects. Ir contributed to the optimized electronic structure of Pd, and the resulting structure has sufficient active sites and accelerated charge transfer. In the overall HSE test, the system sustained operation at 10 mA/cm^2^ under a low voltage of 0.38 V for 15 h, producing acetone with FE of 95.1%.

Despite these results, HSEs face their own constraints that must be addressed before practical deployment. The choice of anodic reaction entails inherent trade‐offs and presents one of the fundamental concerns. In particular, the selection of SMs can be problematic. For sacrificial agents, the thermodynamics is favorable, yet the associated toxicity and regulatory concerns can't be neglected. For feedstock‐type SMs, the products confer added value to the electrolysis, but at the expense of poor selectivity and limiting current densities, as well as poor substrate solubility in the case of biomass. Other than the mechanistic issues, HSEs still require more optimized system design to reduce the energy consumption [203]. Although multi‐stack configurations have been proposed as a solution for lower energy consumption, the concurrently raised internal resistance of the HSE system and the operational stability remain unsatisfactory [206].

### 5.3 Major Challenges of Seawater Electrolyzers

#### 5.3.1 Hindrance to Practical Application

The practical application of electrolyzers in industrial‐level DSE usually operates under Amper‐level current density and needs stable operation for thousands of hours, while, as we discussed before, most of the current DSE electrolyzers focus on lower current density (<500 mA/cm^2^). And the cell voltage is usually significantly higher than pure‐water electrolysis. In alkaline seawater electrolysis, when coupled with natural seawater without a supporting electrolyte, the performance can further decrease. To the best of our knowledge, the lifespan of current DSE electrolyzer (≤3200 h) is at least one magnitude lower than conventional alkaline/pure water electrolyzer (80 000 h in 2026 DOE target), and the performance using natural seawater (<1000 mA/cm^2^ at 2 V) is not even comparable with pure water electrolysis (3000 mA/cm^2^ at 1.8 V). When coupled with the actual marine environment, the lifetime of the current DSE technique can be further affected by the fluctuating ocean conditions [69]. More factors need to be considered for large‐scale DSE than laboratory‐level design, such as the synergistic operation of interconnected industrial electrolyzer stacks, the optimization of mass transport at high current density, and the advanced management of both electrolyte and seawater circulation. Ensuring a uniform electrolyte flow and current distribution may be more useful than developing advanced catalysts. While the development of catalysts with high activity, stability, and selectivity is still important for DSE, a critical trade‐off presents in DSE research development: designing highly robust catalysts or engineering the system to protect current catalysts. In the DSE system with in situ purification functions, less complex and cost‐effective conventional materials can survive as the impurity ions are isolated from the electrolyte.

Except for the catalyst design, the physical design of the electrolyzer architecture is also paramount. Innovations such as 3D‐structured electrodes, optimized PTL, and advanced flow fields can significantly enhance mass transport and facilitate gas bubble removal. Furthermore, minimizing the interfacial resistance between the electrode, electrolyte, and membrane is crucial for improving the overall energy efficiency. The corrosive seawater environment also brings challenges to electrolyzer components, which require corrosion‐resistant materials. Given that different electrolyzer configurations possess distinct advantages and disadvantages, future research must focus on targeted improvement strategies for each specific configuration. This includes advancing electrolyte management systems to constantly handle the precipitation of metal hydroxides or the precipitation of salts. Ultimately, the development of advanced membranes is the most important factor. Achieving superior long‐term stability and high ion selectivity in these membranes is essential to unlock the potential of the DSE system.

Other than electrolyzer architecture, the reliance on an external power grid further complicates the practical deployment of DSE, motivating the exploration of self‐powered systems that couple seawater splitting with renewable sources (solar, wind, hydro) or battery‐based units such as magnesium/seawater and Zn–air batteries [207]. Such systems have demonstrated encouraging hydrogen‐production capabilities. For example, a self‐powered configuration integrating a magnesium/seawater battery with a DSE achieved a hydrogen production rate of up to 12.42 mL/cm^2^/h at a Mg‐H_2_ conversion efficiency of 80.87% [208]. Nevertheless, their full realization remains constrained by the intermittent and unstable current output, the limited durability of materials under natural seawater, and the difficulty of maintaining high activity, stability, and selectivity, leaving energy self‐sufficiency a serious technical challenge for practical application [207].

#### 5.3.2 Economic and Environmental Trade‐Offs

The techno‐economic analysis of seawater electrolysis highlights key challenges associated with its industrialization. Seawater splitting presents unique economic and environmental trade‐offs due to its reliance on complex electrochemical processes in a challenging medium. Corrosion and chlorine competition remain significant barriers to scalability and long‐term system durability, necessitating the development of specialized materials and processes. While life cycle assessments (LCA) and sustainability evaluations have been proposed to address these challenges, the literature lacks comprehensive metrics for environmental impacts, costs, and social implications. The integration of renewable energy sources, such as solar and wind, alongside innovative designs in electrolyzers and catalysts, offers a pathway to improve cost efficiency and sustainability [209]. For example, combining offshore renewable energy resources with DSE is a promising field. Mebarban et al. [171] summarized the recent perspectives of DSE for offshore H_2_, and they reported that the LCOH can reach $1.5/kg excluding electrolyzer expenses, due to the infrastructure and specialized equipment. To reduce LCOH and improve the economic competitiveness of DSE coupled with offshore wind, it is essential to increase the capacity.

**FIGURE 11:**
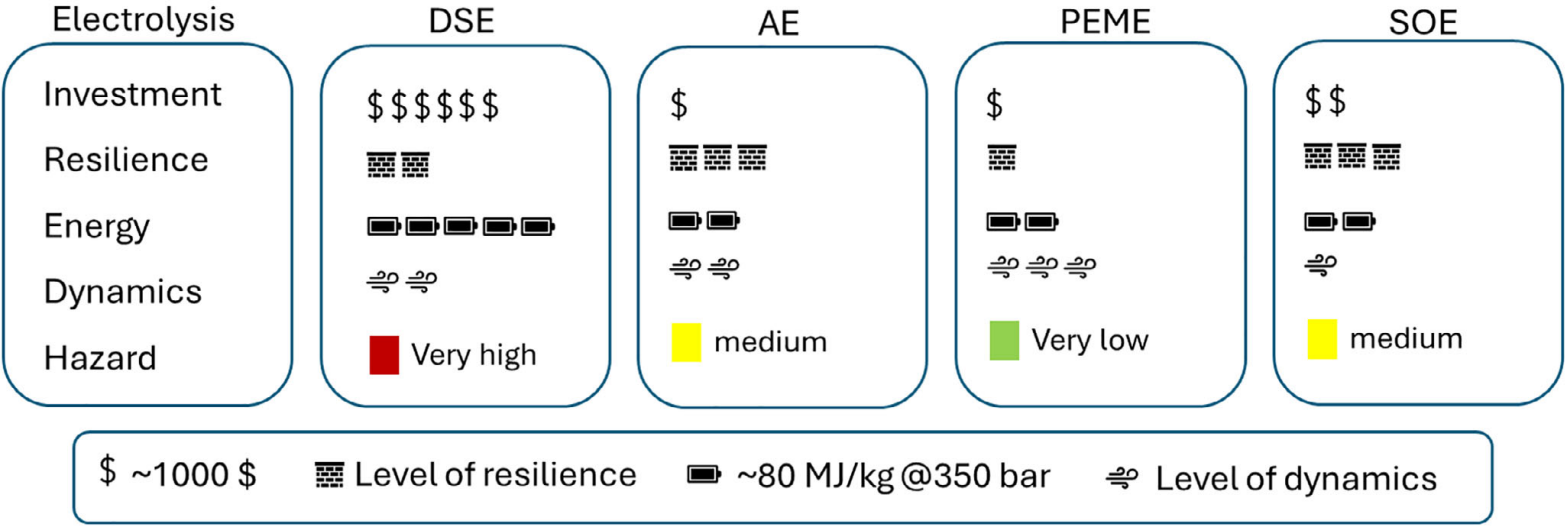
Comparison of attributes of the electrolysis technologies with an asterisk have been estimated; therefore, uncertainty is expected. Adapted with permission [211], Copyright 2020, Elsevier.

For freshwater electrolysis, Aminaho et al.’s research [210] has shown that the electricity cost makes up more than 60% of LCOH for all technologies. When it comes to DSE, the mitigation of chloride‐induced corrosion and the periodic replacement of membranes, catalysts, and structural components in a saline environment, the calculation of LCOH or production cost is calculated differently [211]. The research conducted by d'Amore‐Domenech et al. applies both economic and environmental analyses to evaluate four electrolysis technologies, including DSE. In comparison to commercially mature technologies, such as PEM, seawater electrolysis exhibits both advantages and limitations. For instance, the capital investment for seawater electrolysis exceeds $6000/kW, whereas PEM ranges from $600 to $1300/kW. Similarly, maintenance costs for seawater electrolysis surpass $240/kW/year, compared to the $18–$65/kW/year required for PEME. However, seawater electrolysis demonstrates greater resilience under certain operational conditions. Figure 11 presents a detailed comparison of these electrolysis technologies [211]. Despite the higher LCOH of DSE, recent catalyst‐level advances offer encouraging prospects. Kang's group [40] reported an ultra‐stable corrosion‐resistant RuMoNi catalyst that operates for over 3000 h at 500 mA/cm^2^ in alkaline seawater. When integrated into an AEMWE, the projected cost of the produced H_2_ is $0.85 per gallon of gasoline equivalent (GGE), lower than the 2026 technical target of $2/GGE set by the US, exhibiting good practical potential of the approach. Yi et al. [212] presented another innovative method for descreasingH_2_ production cost. They employed a double‐product strategy that produces green H_2_ together with Mg(OH)_2_ in natural seawater electrolysis with a supersolidophobic Pt catalyst. The LCA shows that this process reduces global warming potential compared to average H_2_ production, generates positive environmental benefit, and can reduce the overall environmental impact.

Overcoming economic and environmental barriers will require coordinated efforts among researchers, policymakers, and industry stakeholders to optimize systems, establish regulatory frameworks, and incorporate environmental performance standards into decision‐making processes. Such approaches are essential to unlock the potential of seawater electrolysis as a viable source of green H_2_ [209].

## 6 Computational and Theoretical Insights

Computational chemistry has been a powerful method to develop mechanistic insights on surface reactions and structure–activity relationships, which complement experimental investigations. Density functional theory (DFT), as a first‐principles approach, is one of the key methods for computing reaction free energies, along with the identification of stable intermediates and predicting catalyst performance. Molecular dynamics (MD) simulations have also been used to better capture realistic operating conditions, which are often simplified in DFT, such as the presence of competing surface reactions, complex electrolyte composition, and temperature effects. Here, the discussion of these methods will be divided into two sections, based on the role of computational methods: mechanistic investigation and catalyst design.

### 6.1 Mechanistic Investigation

**FIGURE 12:**
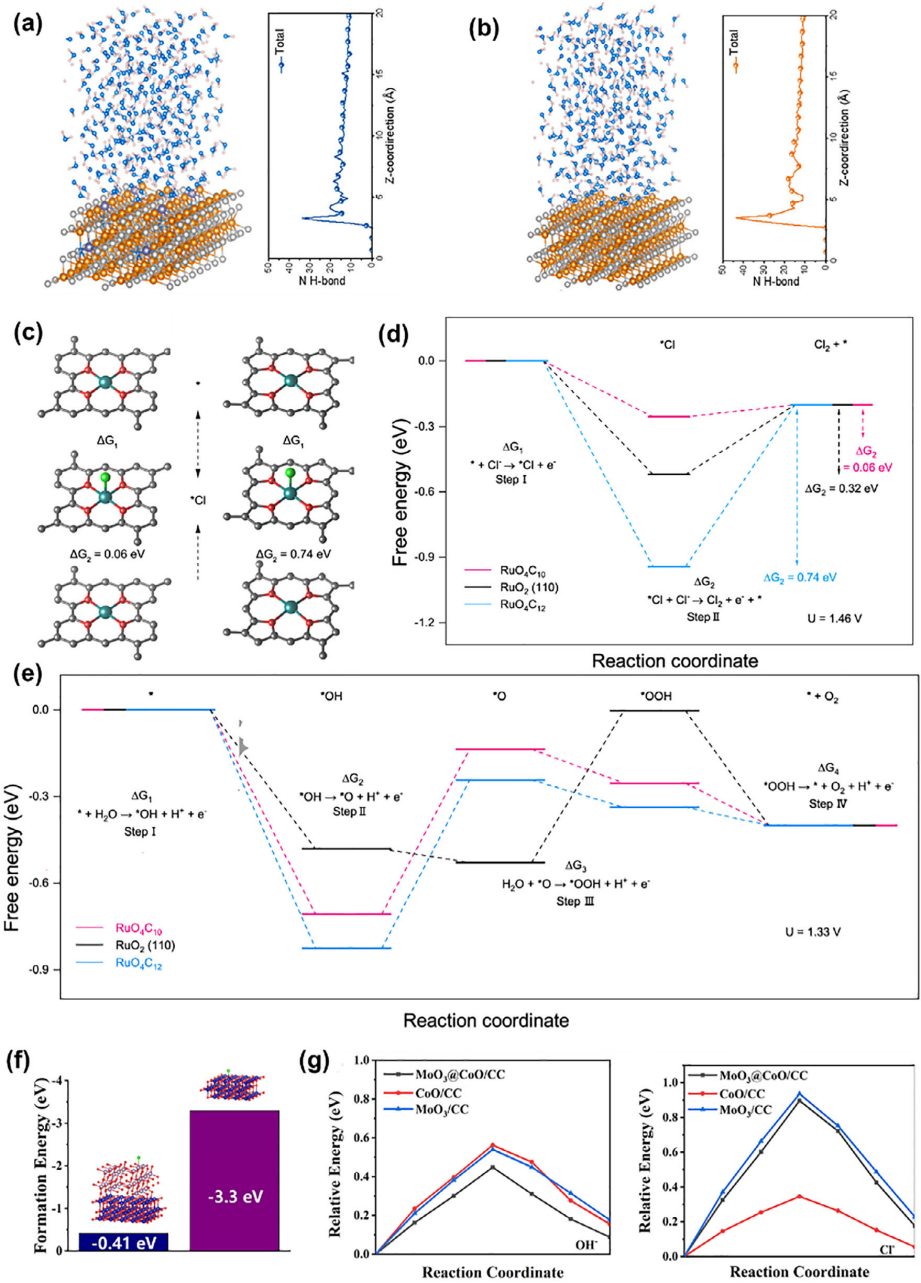
(a,b) The dynamic DFT models of the interface between catalysts and explicit water molecules, along with the number of hydrogen bonds in water as a function of distance from the surface, used for investigating the water affinity toward surfaces. Reproduced with permission [213]. Copyright 2025, John Wiley and Sons. (c) The static DFT models of catalyst (single‐atom catalyst (SAC) sites) used for studying the interaction with intermediate *Cl, along with (d,e) the DFT‐calculated free energy diagrams for ClER and OER. Reproduced under the terms of the CC‐BY license [214]. Copyright 2023, Springer Nature. (f) The adsorption energy of *Cl on different catalysts and (g) the NEB pathway for *OH and *Cl migration within the catalysts [127], used for understanding the effects of surface engineering to ClER and OER. Reproduced under terms of the CC‐BY license [127]. Copyright 2024, Springer Nature.

Complex electrochemistry in seawater electrolysis is expected, with DFT must not only address the conventional HER and OER pathways as in typical water electrolysis, but also the competing reactions originate from Cl^−^ along with the presence of complex solvation environments, as shown in Figure 12a,b. The interaction between water molecules on the surface of catalysts varied depending on multiple factors, such as the intrinsic nature of catalysts, solvation environments (e.g., pH and ions), and electric fields. Figure 12a,b exhibits the correlation between surface composition (Mo‐O‐FeP@IF and FeP@IF, respectively) and water molecules’ distance [213], as reflected by the number of hydrogen bonds. The decreasing number of hydrogen bonds at the interface, due to the polarization effect, resulted to the easier activation of water, formation of intermediates, and consequently the evolution of hydrogen from the surface [213]. Such descriptions have been successfully captured by the use of dynamic DFT, which allows the modelling of explicit water molecules on the surface of catalysts.

The majority of DFT calculations have been used extensively to construct free energy diagrams that were used to investigate the thermodynamic profile of multi‐electron/proton transfer reactions. Figure 12c shows typical models for static DFT calculations [214], which were used to study the reaction coordinates without any description of solvents. The focus is mainly on identifying key intermediates and understanding the potential‐limiting reaction, which are critical parameters for complex systems consisting of multiple competing reactions, such as those in the seawater electrolysis process. Figure 12d,e exhibits the free energy profiles for ClER and OER on transition‐metal single‐atom active sites embedded in nitrogen‐doped carbon supports [214]. The conventional OER pathway follows the sequential formation of adsorbed intermediates of *OH, *O, *OOH, and O_2_. Whereas the ClER pathway follows the formation of adsorbed Cl species with distinct active sites without and with oxygen on the surface, as *Cl and/or *OCl. The calculation of Gibbs free energy of each intermediate as a function of electrode potential (as referenced to SHE) and pH can provide insights into thermodynamics, particularly the potential‐limiting reaction. The following Equation (6) can be used for considering the effect of pH, chloride activity ($a^{Cl^{-}}$) and electrode potential (V vs. SHE).

(6) $$\Delta G=\Delta G^{o}\;+\;RTlnQ\;-\;nFE$$

where $\Delta G^{\circ }\;\;=\;\Sigma_{i}\;\mu_{i}^{\circ }$ is the standard Gibbs energy for the reaction, $Q=\Pi_{i}\;a_{i}^{v_{i}}$ is the reaction quotient, n is the number of electrons transferred, E is the electrode potential referenced to the same zero as Δ*G*°.

**FIGURE 13:**
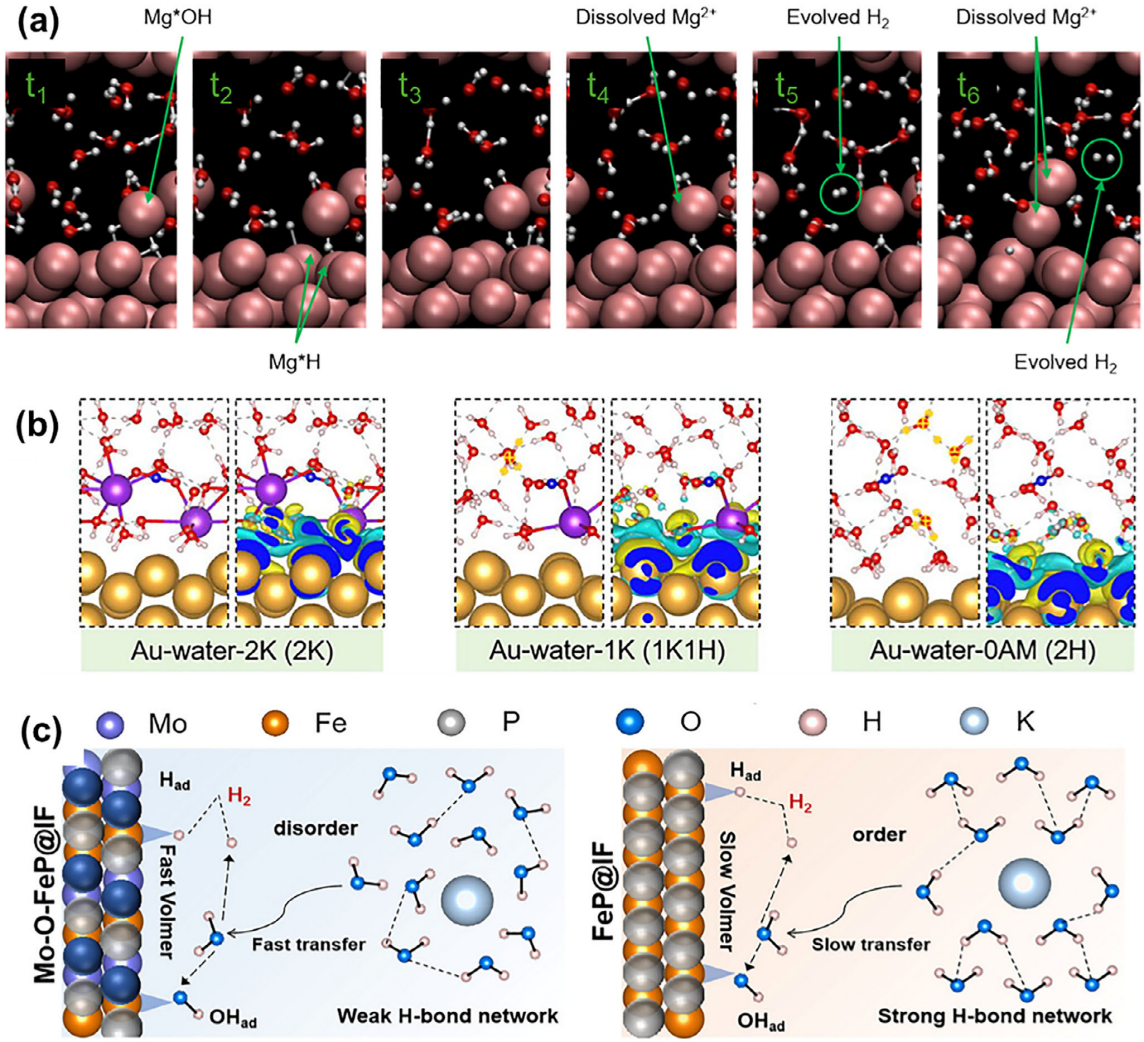
(a) The screenshots of dynamic DFT models of interface with explicit solvent molecules, showing the occurrence of multiple reactions over simulation times. Reproduced under terms of the CC‐BY license [215]. Copyright 2019, Elsevier. (b) The static DFT models of the interface between catalysts and single water molecules, used for calculating the adsorption energy of water molecules, as a descriptor for investigating the water affinity toward surfaces. Reproduced with permission [216]. Copyright 2024, American Chemical Society. (c) Schematic illustrations exhibit the synergistic contributions among the catalyst's surface, water‐molecule network, and cation's presence at the interface on the HER performance. Reproduced with permission [213]. Copyright 2025, John Wiley and Sons.

Upon the identification of key intermediates, the DFT‐calculated adsorption energies can be used as the descriptor that can be further used for the search of better catalysts. A typical approach used is to evaluate the adsorption energy of chloride on the surface, giving insights on how to suppress the parasitic ClER, as shown in Figure 12f. The interaction between catalyst and Cl^−^ can be minimized by modifying the surface, such as by introducing a MoO_3_ layer on top of CoO, which decreases the adsorption energy of chloride [127]. This surface modification strategy is expected to favor the OER over ClER across active sites, which can be better understood using DFT. Figure 12g and Figure 13a show the investigations of kinetic barriers for the migration of OH^−^ and Cl^−^ on the surfaces. The nudged elastic band (NEB) is one of the common methods used to calculate minimum energy paths and transition states for elementary steps, such as H─OH bond breaking, O─O bond forming and Cl─Cl bond forming. NEB in Figure 12g,h gives information on the activation energy involving catalyst‐adsorbate bond‐breaking/forming [127], which can be integrated further into each elementary step in the free‐energy diagrams of OER and ClER, and provides insights into the rate‐limiting reaction. The implementation of DFT has been beneficial for obtaining the thermodynamic and kinetic profiles of interfacial reactions, despite the simple use of a physical model in the calculations. Nonetheless, it is important to note that most calculations neglect the effects of solvents, while some improvements can be made to incorporate them by treating the solvents either implicitly or explicitly. The implicit solvent treatment, as the simplest approach, uses an additional contribution from a continuum dielectric to approximate solvent effects, changing the total electronic energy from DFT. Whereas the explicit solvent treatment, as shown in Figure 12a, introduces solvent molecules at the interface, allowing more rigorous interactions between solvents and catalysts along with the formation of intermediates due to their interactions, as shown in Figure 13a.

(7) $$\begin{array}{ccc} & & \Delta G\;\left(E_{applied},pH,\left[Cl^{-}\right]\right)=\;\;\Delta G_{DFT-vac}-nF\left(E_{applied}-\frac{E_{SHE}^{abs}}{F}\right)\\ & & -m\times 0.0592eV\times pH+p\times 0.0257eV\cdot ln\left[Cl^{-}\right] \end{array}$$

The recent advancements of dynamic DFT and machine learning, as well as the increasing of computing power, allow the complete investigation of elementary steps during electrochemical reactions using larger supercell sizes (a greater number of atoms) and longer simulation times. This also means that measurable outcomes, such as understanding possible reaction coordinates, identifying stable intermediates (and their configuration), and computing energy barriers, can be obtained simultaneously without any pre‐determined assumptions of certain reaction coordinates and intermediates configuration. Figure 13a shows screenshots of dynamic DFT calculations, revealing the occurrence of multiple reactions during simulation times of water dissociation, *H and *OH adsorptions, dissolution of surface atoms, and H_2_ evolution at the interface [215]. The simulations consider the effects of temperature and solvents at the same time, without the needs of adding corrections, making dynamic DFT a more competitive advantage over static DFT as well as traditional force‐field MD calculations to determine potential/rate limiting reaction. Dynamic DFT provides a time‐resolved atomistic view of dynamic reactions at the interface, which gives a better understanding of how the reactions progress under more realistic conditions. The combination with machine learning potentials (MLPs) will also open new opportunities to extend the simulation to a much larger size and longer simulation time, as they allow prediction of forces and energies with near‐DFT accuracy but with a relatively low computational cost.

In addition, the explicit modelling of water molecules at the interface can also be used to further investigate the influence of cations on HER performance. Figure 13b shows the changes in the local concentration of water molecules at the interface due to the presence of cations, which alter the water–water interactions and water rotational dynamics as well as facilitate the charge transfer process. As the cation's radius and valence increase, the coordination number between cation and water also increases, making interfacial water more available on the surface for the HER. The synergistic contribution, as shown in Figure 13c, exhibits the influence of both the catalyst's surface and the cation in forming a microenvironment for HER, allowing either faster or slower hydrogen recombination by altering the behavior of water–water interactions at the interface [213]. Such information is critical for investigating seawater electrolysis performance under different catalysts, as the presence of impurities (e.g., multiple cations) significantly influences the HER performance, which can now be better understood using dynamic DFT simulations taking into account different factors.

In regard to the generation of H_2_, DFT has also been used to study the HER mechanisms, as shown in Figure 13b,c. The investigations of adsorption energy of single water molecules along with the free energy diagram for HER were presented. Strong water adsorption energy on the catalyst is revealed as one of the indicators for facilitating subsequent water dissociation and HER [213]. Meanwhile, for HER, the catalyst ideally should exhibit relatively neutral affinity toward hydrogen adsorption to minimize a large potential‐limiting reaction for HER. It is shown in Figure 13c that the *H adsorption energy in FeP is the strongest, followed by those in Mo─O─FeP with Fe as the active site and Pt [213]. The adsorption of *H is not favorable at the P active site of Mo─O─FeP, as reflected by the positive energy. Nonetheless, in the view of HER, the Fe active site in Mo─O─FeP and Pt shows the most ideal case, with the lowest potential‐limiting reaction for hydrogen desorption/evolution.

### 6.2 Catalysts Design

**FIGURE 14:**
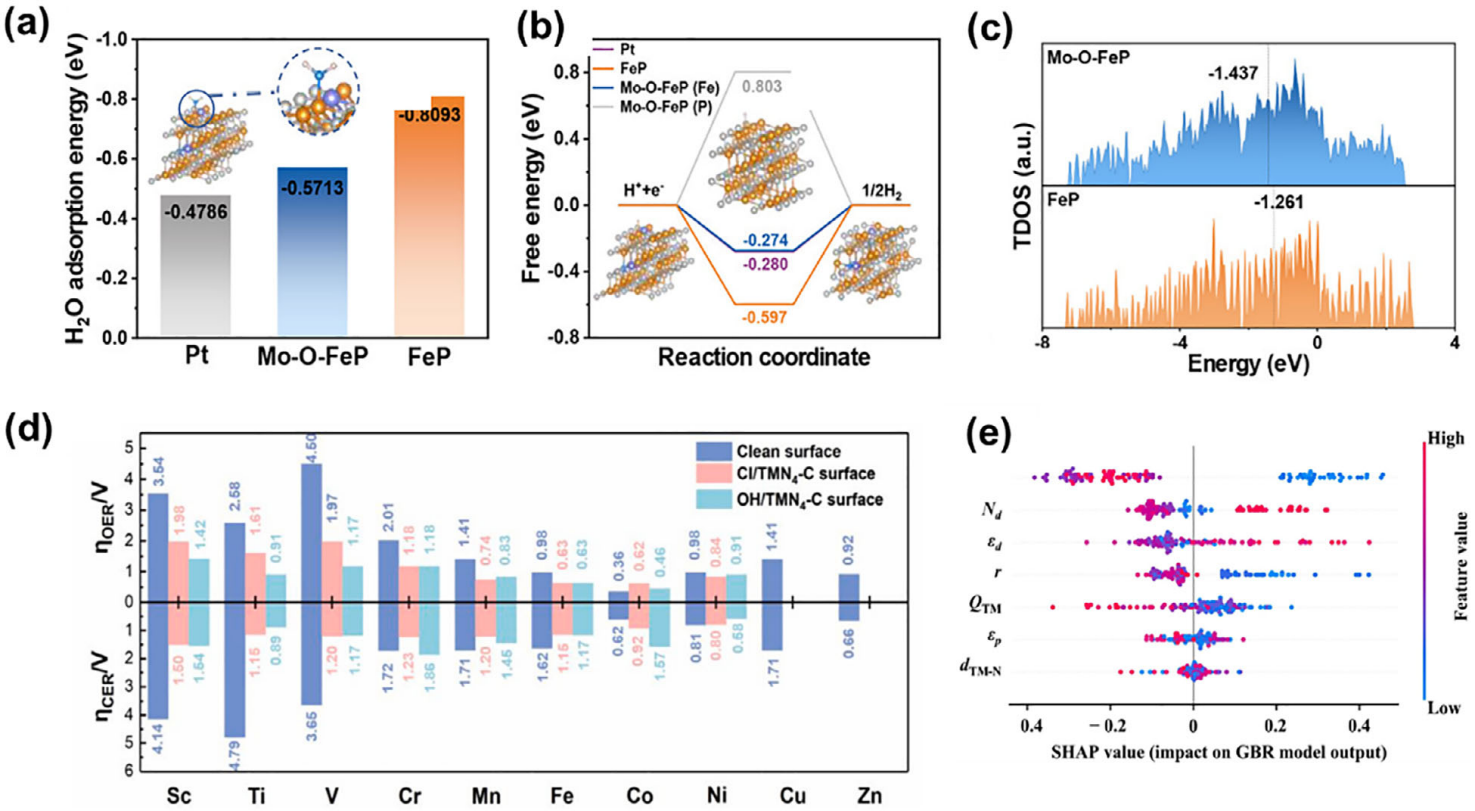
(a) Free energy diagram for HER on different catalysts [213]. (b) The density of states [213], one of the important electronic properties, as calculated from the DFT that is used to develop the d‐band center. (c) The density of states, one of the important electronic properties, as calculated from the DFT that is used to develop the *d*‐band center. Reproduced with permission [213]. Copyright 2025, John Wiley and Sons. (d) DFT‐calculated overpotentials for ClER and OER for different TM centers used in SACs [217], which are used to aid the screening of ideal TMs. Reproduced with permission [217]. Copyright 2023, Elsevier. (e) SHAP summary diagram showing the importance value of seven features for predicting OER overpotential using a GBR model [218], leading to the identification of best catalysts with excellent OER selectivity and activity. Reproduced with permission [218]. Copyright 2026, Elsevier.

In addition to the investigation of atomic mechanisms at the interface for HER, OER, and ClOR, computational methods based on DFT have also been used for predicting the ideal catalysts, such as through high‐throughput calculations and catalyst comparison. The screening of materials is made possible through descriptor‐based design strategies. One important descriptor is the d‐band center position, which measures the distance between the d‐band center and the Fermi level. This feature can be easily calculated from the DOS obtained using DFT. Figure 14a exhibits the DOS profile of different catalysts, with Mo‐O‐FeP having a d‐band center much further from the Fermi level than FeP, which translates to a better HER performance [213]. The electronic structure d‐band center, along with other descriptors, such as adsorption energies for key intermediates, are important descriptors that can be used to guide the design and/or discovery of better catalysts for different reactions. The particular example is the search for transition metals of single‐atom catalysts for seawater electrolysis, as shown in Figure 14b. DFT‐guided design has enabled the investigation of overpotentials of multiple transition metal (TM) elements for facilitating OER and ClER [217]. Lower overpotential (potential‐limiting reaction) for a given reaction suggests higher catalytic activity for the reaction. Whereas the comparison of OER and ClER overpotentials provides information on the competitiveness of two reactions thermodynamically, guiding the search for catalysts with higher OER selectivity and activity than ClER. Such knowledge can aid experiments to synthesize and characterize suitable catalysts in a more time‐efficient and cost‐effective approach. From Figure 14b, the search for TM based on the overpotential criteria revealed an observation of trend, in which with the increase of valence electrons, both overpotentials for OER and ClER decrease from Sc to Co, whereas the trend then is reversed starting from Ni [217]. Upon the evaluation of overpotentials, the identification of MnN4‐C, FeN4‐C, and CoN4‐C as ideal catalysts for seawater electrolysis is made [217].

The integration of high‐throughput DFT with machine learning (ML) also exhibits potential for reducing the cost of exploring large compositional spaces of catalysts and enabling the use of feature engineering to better select the catalysts. Figure 14c exhibits the SHAP summary diagram for the 144 SACs investigated, showing the importance of different features, that is, the metal's electronegativity (*χ*), the metal's Bader charge (QTM), the d‐band center of metal atom (*εd*), the number of d‐electrons of the metal atom (Nd), the metals’ atomic radius (*r*), the average distance between metal atom and coordinating N atom (dTM‐N), and the p‐band center of the coordinating N‐atom (*εp*), to the gradient boosting regression (GBR) prediction of OER overpotential [218]. It is revealed that weaker *χ*, smaller *r*, and more Nd have positive SHAP values, which leads to the identification of four SACs with the best combination of OER selectivity and activity using TMs of Co, Rh, and Ir [218].

Although most studies on the search for better catalysts mostly focused on SAC, due to its relatively simple model for DFT, the use of ML can be implemented to unravel design strategies for more complex catalyst systems, such as high‐entropy alloys [71], LDHs [72, 124], and metal oxides [22, 219], by using datasets obtained from experiment and theory. These materials have been previously investigated and shown promising seawater electrolysis performance, and yet the design principles are not fully understood.

## 7 Conclusions and Future Outlooks

Realizing the practical implementation of seawater electrolysis requires a decisive shift from laboratory optimization toward scalable system design. Addressing scalability challenges requires the development of cost‐effective and industrially viable strategies that cover materials design, electrode architectures, and device fabrication. Beyond catalyst activity, future research should emphasize synthesis routes and fabrication processes that are compatible with large‐scale manufacturing, including simplified, one‐pot sustainable synthesis methods and the use of earth‐abundant elements. The rational design of active materials with scalable electrode architectures will be essential to reduce production complexity and system costs while maintaining high performance.

Enhancing catalyst stability in real seawater conditions is another critical area of focus. Long‐term durability in the presence of biofouling, chloride‐induced corrosion, and contamination from impurities must be ensured to meet industrial standards. To this end, the development of protective coatings on electrolyzers, such as hydrophobic or anti‐corrosive layers, and incorporating elements like phosphorus or nitrogen to enhance corrosion resistance, could improve corrosion resistance. Additionally, optimizing catalyst microstructures to prevent degradation and maintain performance over extended periods will be essential for industrial applications.

In addition to catalyst design, seawater‐compatible electrolyzers must become a central research focus. Conventional AWE, PEMWE, and AEMWE systems suffer from membrane degradation, chloride crossover, scaling, and corrosion under real seawater conditions. Future DSE development should therefore prioritize device‐level design, including selective and anti‐fouling membranes, corrosion‐resistant components, optimized flow management, and stable high current density operation over thousands of hours. Bridging laboratory demonstrations and industrial deployment will ultimately depend on integrated system engineering rather than catalyst optimization alone.

The lack of standardized testing protocols remains a major barrier to consistent evaluation and benchmarking across different studies. Future work should focus on establishing universally accepted protocols for electrolyte composition, including specifying whether simulated or natural seawater is used and detailing its treatment and composition. Standardizing current densities for activity and cataloguing catalyst performances under these standardized conditions can accelerate innovation and collaboration in the field.

Alongside physics‐based simulation, data‐driven approaches represent a particularly promising avenue for accelerating DSE catalyst discovery. The integration of high‐throughput DFT with ML has already demonstrated its value in efficiently navigating large compositional spaces, with feature‐importance analyses such as SHAP revealing which electronic and structural descriptors most strongly govern OER selectivity over the competing ClER. Extending these strategies beyond the SACs to more complex and industrially relevant catalysts, such as HEAs, LDHs, and mixed metal oxides, could unlock design principles that remain poorly understood at present. Moreover, the coupling of MLPs with dynamic DFT offers a route to simulate realistic seawater interfaces at length and time scales far beyond conventional first‐principles methods. The continued development of shared experimental and computational stability measurements will enable more consistent benchmarking. Developing comprehensive databases of datasets, alongside standardized descriptors, will be essential to fully realize the potential of these tools and to boost the design of robust DSE catalysts.

Looking ahead, the integration of seawater electrolysis systems with renewable energy sources such as solar and wind will be pivotal. Research should focus on designing catalysts and electrolyzer systems that can efficiently operate under variable power inputs characteristic of renewable energy. Developing adaptable system architectures and control strategies will enhance overall energy efficiency and reliability. Furthermore, comprehensive techno‐economic analyses and LCA should be conducted to optimize system designs, reduce operational costs, and ensure environmental sustainability, thereby promoting the economic viability of large‐scale green H_2_ production from seawater.

Finally, overcoming economic and policy‐related barriers will require coordinated efforts among policymakers and industry stakeholders, and the research community. These include implementing regulatory frameworks, providing economic incentives for research and industrial adoption, and fostering public–private partnerships to advance technology readiness levels and pave the way for commercial‐scale deployment.

## Conflicts of Interest

The authors declare no conflicts of interest.

## Data Availability

Data sharing not applicable to this article as no datasets were generated or analyzed during the current study.
